# Associations of habitual coffee intake with testosterone and cardiometabolic markers: the Northern Finland birth cohort 1966 study

**DOI:** 10.1007/s00394-026-04038-z

**Published:** 2026-07-16

**Authors:** Luca Verroest, Jari Jokelainen, Shalini Choudhary, Jaroslaw Walkowiak, Toni Karhu, Saranya Palaniswamy, Juha Auvinen, Marjo-Riitta Jarvelin, Karl-Heinz Herzig, Ghulam Shere Raza

**Affiliations:** 1 Research Unit of Biomedicine and Internal Medicine, Faculty of Medicine, University of Oulu, Oulu, Finland; 2 Arctic Biobank, Infrastructure for Population Studies, Faculty of Medicine, Northern Finland Birth Cohorts, University of Oulu, Oulu, Finland; 3 Pediatric Gastroenterology and Metabolic Diseases, Pediatric Institute, Poznan University of Medical Sciences, Poznan, Poland; 4 Center for Life Course Health Research, Faculty of Medicine, University of Oulu, Oulu, Finland; 5 Department of Epidemiology and Biostatistics, School of Public Health, Imperial College London, London, UK; 6 Biocenter Oulu, University of Oulu, Oulu, Finland; 7 Unit of Primary Care, Oulu University Hospital, Oulu, Finland; 8 Department of Life Sciences, College of Health and Life Sciences, Brunel University London, Uxbridge, UK; 9 Research Unit of Population Health, University of Oulu, Oulu, Finland; 10 Medical Research Center, Oulu University Hospital, Oulu, Finland

**Keywords:** Coffee, Metabolomics, Testosterone, Body composition, Glucose metabolism, Nutritional epidemiology

## Abstract

**Purpose:**

Coffee is a widely consumed source of bioactive compounds globally, yet its associations with circulating metabolites, sex hormones, and cardiometabolic markers remain incompletely characterised. We investigated these associations in a large population-based cohort, with particular attention to sex-specific hormonal profiles and metabolomic signatures.

**Methods:**

Cross-sectional data from 2,264 participants (47% men) of the Northern Finland Birth Cohort 1966 (NFBC1966) at age 46 were analysed using sex-stratified Spearman correlations and multivariable linear regression models adjusted for BMI, educational attainment, smoking, physical activity, and alcohol intake.

**Results:**

Higher coffee intake groups showed lower total and visceral fat and higher skeletal muscle mass, while BMI was comparable across groups. Inverse correlations were observed between coffee intake and circulating branched-chain amino acids (BCAAs) in both sexes. In men, higher coffee intake correlated with a more favourable glucose–insulin profile. Multivariable-adjusted models revealed a distinct hormonal pattern: positive associations with total testosterone (β = +0.29 nmol/L per cup/day), bioavailable testosterone, and sex hormone–binding globulin (SHBG), alongside inverse associations with free testosterone and the free androgen index (FAI). In women, hormonal associations were more limited, with positive associations for SHBG and inverse associations for free testosterone and FAI.

**Conclusions:**

Higher habitual coffee intake groups showed a leaner body composition, correlated inversely with circulating BCAAs in both sexes, and were independently associated with a sex-specific hormonal profile that may be metabolically relevant, particularly in men. Future longitudinal and intervention studies are needed to establish causality and identify the coffee-derived bioactive compounds involved.

**Supplementary Information:**

The online version contains supplementary material available at 10.1007/s00394-026-04038-z.

## Introduction

Coffee is one of the most widely consumed beverages globally and a defining component of daily life across many populations. In Europe, average per capita consumption is approximately 5.7 kg per year, accounting for nearly one quarter of global coffee intake. Finland stands out as the highest coffee-consuming country worldwide, with an annual intake of approximately 11.8 kg per capita, while other Nordic countries also rank among the highest consumers (European Coffee Report 2022/2023). Beyond its widespread consumption, coffee is deeply embedded in daily routines and is often consumed for stimulation, comfort, social connection, mood enhancement, and stress management [1].

Apart from its cultural and psychological relevance, coffee is a chemically complex beverage with over a thousand bioactive compounds, including alkaloids such as caffeine and trigonelline, diterpenes like cafestol and kahweol, and phenolic compounds such as chlorogenic acids [2, 3]. Many of these constituents possess antioxidant and anti-inflammatory properties, which may help protect against oxidative-stress–related conditions. Consistent with these mechanisms, reviews report inverse associations between coffee intake and several cardiometabolic outcomes, including metabolic syndrome (MetS), type 2 diabetes (T2D), coronary artery disease, stroke, and reduced all-cause mortality [4–6]. Furthermore, coffee consumption has been associated with lower overall cancer risk, supported by evidence indicating reduced risks across a range of cancer types [7–10]. Across these outcomes, reported harmful associations are generally weak and are largely attenuated or nullified after adjustment for smoking. Although potential risks of coffee consumption include high-dose caffeine toxicity, interactions with certain medications, processing-related contaminants, and increases in total and low-density lipoprotein (LDL) cholesterol due to diterpenes in unfiltered coffee [11, 12], the overall evidence indicates that moderate consumption is safe for most adults. Intake levels of up to 400 mg caffeine per day are not associated with overt adverse effects [13], and epidemiological studies consistently report the greatest risk reductions for multiple health outcomes at approximately three to four cups per day, suggesting that habitual coffee consumption is more likely to confer health benefits than harm [7]. The physiological effects of coffee may nevertheless vary depending on the specific profile and concentration of bioactive compounds, which are influenced by factors such as bean origin, roasting, and brewing method [14, 15].

In addition to these cardiometabolic effects, sex hormones may represent an important biological pathway linking coffee consumption to metabolic health. Testosterone and sex hormone-binding globulin (SHBG) are key regulators of adiposity, insulin sensitivity, and vascular function, and have been implicated in the development of MetS, T2D, and cardiovascular disease [16]. However, evidence on dietary determinants of these hormonal pathways, including coffee consumption, remains limited and inconsistent.

Although coffee consumption has been associated with a range of favourable health outcomes, the biological mechanisms underlying these effects remain incompletely understood. Recent reviews highlight that roasting and brewing methods, interindividual genetic variation, and lifestyle factors such as smoking may modify physiological responses to coffee [4–6, 17], emphasizing the need for studies that examine metabolic and cardiometabolic markers in greater depth. In contrast, effects of coffee consumption on hormonal outcomes including testosterone have been examined far less frequently, and existing studies report variable findings [18–21]. The objective of this cross-sectional study was to examine habitual coffee intake in relation to circulating metabolites, cardiometabolic, and hormonal markers in adults participating in the Northern Finland Birth Cohort 1966 (NFBC1966) study.

## Methods

### Study population

Data for the present study were drawn exclusively from the 46-year follow-up (2012) of the NFBC1966, a population-based cohort established in the provinces of Oulu and Lapland, in Finland [22, 23]. The cohort comprises children with expected dates of birth between 1 January and 31 December 1966. In total, 12,058 live-born children (96.3% of all births in the region that year, according to Statistics Finland) were enrolled and have been prospectively followed from the prenatal period onwards [24].

Follow-up examinations were conducted at ages 1, 14, 31, and 46 years using a combination of questionnaires, clinical assessments, and linkage to national health and administrative registers. At the 46-year follow-up in 2012, all cohort members living in Finland with a known address (*n* = 10,331) were invited to participate. Data collection comprised four self-administered questionnaires covering background, lifestyle and health; economic situation and working life; opinions and experiences; and supplementary health-related questions, including detailed information on diet, smoking, alcohol use, physical activity, medication use, and diagnosed diseases. In total, 7,146 participants returned at least one questionnaire, and 5,832 attended the clinical examination conducted by three trained nurse teams across 36 locations in Finland, using standardized protocols.

Participants were instructed to fast for 12 h overnight and to abstain from smoking and coffee prior to the clinical examination. The examination included anthropometric measurements, blood pressure assessment, and an oral glucose tolerance test (OGTT). Fasting biological samples (blood) were collected. All participants provided written informed consent. The study was approved by the Ethical Committee of the Northern Ostrobothnia Hospital District, Oulu, Finland (EETTMK 94/11, 17.09.2012), and conducted in accordance with the Declaration of Helsinki.

**Fig. 1:**
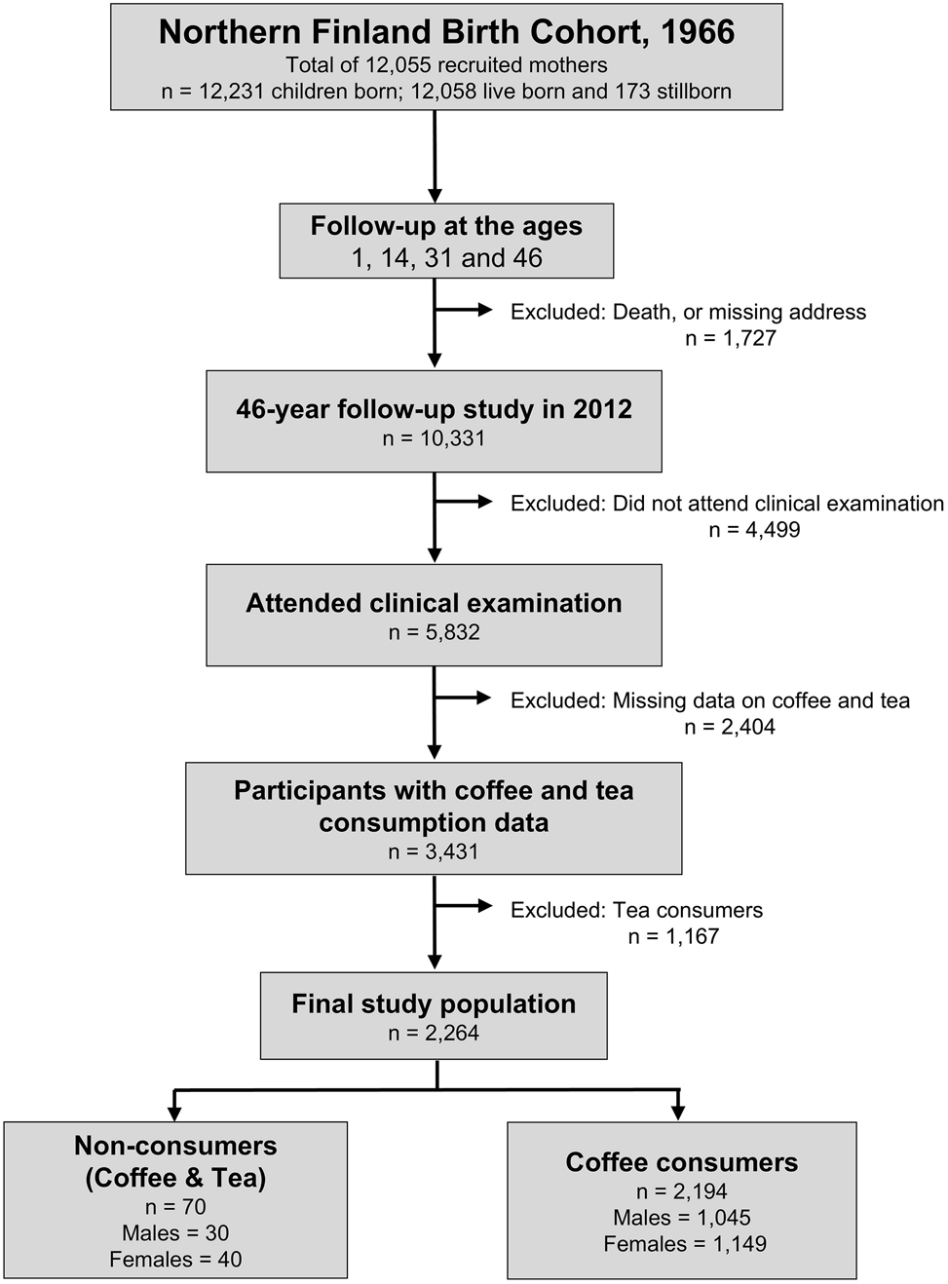
Flowchart of participant selection for the present study

For the current analyses, participants with missing information on coffee and tea intake were excluded. To reduce exposure heterogeneity and potential confounding, we restricted the sample to individuals who reported consuming coffee only or neither coffee nor tea. The final analytical sample comprised 2,264 participants, including 2,194 coffee consumers and 70 non-consumers. The distribution of habitual coffee intake, stratified by sex, is shown in Supplementary Figure S1. Participant selection and exclusion criteria are summarized as flowchart (Fig. 1).

### Assessment of coffee consumption

Coffee consumption was assessed using a self-administered postal questionnaire. Participants were asked: “How many cups on average a day and how many years have you drunk coffee and/or tea?”. Response fields were provided for filtered coffee, boiled coffee, cappuccino, and tea. For the present analyses, habitual coffee intake was calculated as the total number of coffee cups consumed per day and expressed as cups/day. The questionnaire did not define the volume of one cup, and no visual aid, such as cup-size pictures, were provided. For descriptive analyses, participants were classified into four categories: non-consumers (0 cups/day), low consumers (1–2 cups/day), moderate consumers (3–4 cups/day), and high consumers (≥ 5 cups/day). These categories were applied in the baseline characteristics tables, while coffee intake was treated as a continuous variable in correlation and regression analyses. Coffee type was available for 2,173 coffee consumers (96.0% of the analytical sample). Among these consumers, filtered coffee was most common (97.8%), followed by boiled coffee (13.5%) and cappuccino (3.1%), with multiple responses allowed. Information on bean type, roast level, detailed brewing characteristics, and additions such as milk or sugar was not collected.

### Clinical and laboratory measurements

Anthropometric assessments at the 46-year examination included height (cm), weight (kg), and waist and hip circumference (cm), from which BMI (kg/m^2^) and waist-to-hip ratio (WHR) were calculated. For descriptive analyses, BMI was additionally categorized according to World Health Organization (WHO) criteria as underweight (< 18.5 kg/m^2^), normal weight (18.5–24.9 kg/m^2^), overweight (25.0–29.9 kg/m^2^), and obese (≥ 30.0 kg/m^2^) [25]. Body fat percentage, visceral fat area, and skeletal muscle mass were measured using bioimpedance analysis (BIA). Blood pressure was recorded three times using an automated oscillometric device (Omron M10-IT, Kyoto, Japan) and mean systolic and diastolic values were used.

Fasting plasma glucose (FPG) and fasting serum insulin (FSI) were used to calculate fasting indices of insulin resistance, β-cell function, and insulin sensitivity. Insulin resistance was estimated using the homeostatic model assessment for insulin resistance (HOMA-IR; FPG × FSI / 22.5), β-cell function using the HOMA2-β index ((20 × FSI) / (FPG − 3.5) × 100), and insulin sensitivity using the Quantitative Insulin Sensitivity Check Index (QUICKI), calculated as 1 / (log FPG + log FSI). The standard 2-hour OGTT was performed, with plasma glucose and serum insulin measured at 0, 30, 60, and 120 min following ingestion of 75 g glucose. OGTT data were used to calculate glucose and insulin areas under the curve (AUC).

Serum total cholesterol, high-density lipoprotein (HDL), low-density lipoprotein (LDL), and triglycerides were measured using enzymatic assays. High-sensitivity C-reactive protein (hsCRP) was assessed by an immune nephelometric assay (BN ProSpec, Siemens Healthcare Diagnostics Inc., Newark, DE, USA) and categorized as < 1, 1–3, or ≥ 3 mg/L. MetS was defined according to the International Diabetes Federation criteria [26]. 10-year cardiovascular disease risk was additionally estimated using the FINRISK score, an estimate incorporating age, smoking status, blood pressure, lipid levels, diabetes status, and family history of cardiovascular disease [27].

Hormonal analyses included serum total testosterone and SHBG, measured using LC-MS/MS (Agilent Technologies, Wilmington, DE) and electrochemiluminescence immunoassay (Immulite 2000; Siemens Healthcare, Llanberis, UK), respectively. Free testosterone was calculated using mass-action equations and the free androgen index (FAI) as (testosterone / SHBG) × 100.

### NMR metabolomics

Fasting serum samples were analysed using a high-throughput proton nuclear magnetic resonance (NMR) metabolomics platform (Nightingale Health Ltd., Helsinki, Finland). This platform enables simultaneous quantification of 164 lipid and metabolite measures, including a panel of 14 lipoprotein subclasses with their lipid constituents (triglycerides, phospholipids, free and esterified cholesterol), a wide range of fatty acid fractions, apolipoproteins, amino acids, glycolysis-related metabolites, ketone bodies, and several inflammation-related markers [28].

### Statistical analyses

Baseline characteristics were summarized across categories of coffee consumption. Continuous variables were described as mean ± SD, and group differences were assessed using Welch’s one-way analysis of variance (ANOVA). Categorical variables were presented as counts and percentages and compared using Pearson’s χ^2^ tests; for sparse multi-level variables, overall *p*-values were estimated using Monte Carlo simulation (10,000 replicates).

The distribution of continuous variables was assessed visually using histograms and Q–Q plots for representative variables across outcome domains. Several variables showed non-normal distributions; therefore, non-parametric methods were used where appropriate.

Correlations between coffee intake and circulating metabolites and cardiometabolic markers were first evaluated using Spearman rank correlations, stratified by sex. To account for multiple testing across the metabolomic markers, *p*-values were adjusted using the false discovery rate (FDR) according to the Benjamini–Hochberg procedure. Volcano plots were used to visualize the strength (ρ) and significance of these associations across the metabolomic and cardiometabolic panels.

Serum total testosterone concentrations were compared across coffee intake groups separately for men and women using non-parametric Wilcoxon rank-sum tests. Results were visualized with sex-stratified boxplots to illustrate the distribution and dose-related pattern.

Covariates were selected a priori as potential confounders based on biological plausibility and established associations with habitual coffee intake and cardiometabolic and hormonal outcomes. Accordingly, the adjusted models included the following anthropometric, sociodemographic, and lifestyle factors: BMI, educational attainment at age 46 (basic or less, secondary, tertiary), smoking status (never; former—quit > 6 months prior to the clinical examination; former—quit ≤ 6 months prior to the clinical examination; current), alcohol consumption (average drinks/week), and leisure-time physical activity assessed by questionnaire and categorized into four levels reflecting increasing intensity and volume of activity, ranging from predominantly sedentary behaviour to regular competitive training.

To further evaluate independent associations, sex-stratified multivariable linear regression models were fitted for each hormonal marker with coffee intake (cups/day) as the main predictor. Model 1 was unadjusted; Model 2 was adjusted for BMI; and Model 3 was additionally adjusted for educational attainment, smoking status, physical activity, and alcohol consumption. Participants were excluded on a per-analysis basis if data on the outcome or covariates were missing; no imputation was performed. For female hormonal analyses, women with self-reported PCOS or missing PCOS status were excluded because of the known influence of PCOS on androgen-related markers. Specifically, women who reported a PCOS diagnosis (4.7%) or did not provide an answer (6.2%) were excluded from these analyses.

Sensitivity analyses were performed to assess the robustness of the findings. First, anthropometric and body composition comparisons across coffee consumption groups were repeated after excluding participants with extreme BMI values (< 18.5 or > 40 kg/m^2^). Second, all regression models were repeated after excluding participants using lipid-modifying or anti-hypertensive medications, with no material changes in the results. Exploratory generalized additive models (GAMs) using a smooth term for coffee intake showed no meaningful improvement over linear models for most hormonal outcomes; therefore, linear regression was retained as the primary approach.

Results are presented as correlation coefficients (ρ) for the non-parametric analyses and β-estimates with 95% confidence intervals (CI) for the regression models. All analyses were conducted in R (version 4.5.1), with a two-sided *p* < 0.05 considered statistically significant. This study was reported in accordance with the Strengthening the Reporting of Observational Studies in Epidemiology (STROBE) guidelines for cohort studies.

## Results

### Study population

The final sample comprised 2,264 participants from the 46-year follow-up of the NFBC1966, including 2,194 coffee consumers and 70 non-consumers. Coffee consumers were further divided into low (1–2 cups/day, *n* = 296), moderate (3–4 cups/day, *n* = 822), and high (≥ 5 cups/day, *n* = 1,076) intake groups. Baseline sociodemographic, lifestyle, anthropometric, and body composition characteristics by coffee consumption group are summarized in Table 1. Overall, 47% of participants were male. The proportion of men increased across categories of coffee consumption, from 35% in low consumers to 55% in high consumers (*p* < 0.001).

**Table 1:** Participant characteristics by categories of habitual coffee consumption

Measure	Overall	Non-consumers	Low (1–2 cups/d)	Moderate (3–4 cups/d)	High (≥ 5 cups/d)	*p*-value
	*N* = 2,264	*N* = 70	*N* = 296	*N* = 822	*N* = 1,076	
*Sociodemographics & lifestyle*						
Sex						< 0.001***
Men	1,075 (47%)	30 (43%)	105 (35%)	351 (43%)	589 (55%)	
Women	1,189 (53%)	40 (57%)	191 (65%)	471 (57%)	487 (45%)	
Education level						< 0.001***
Basic or less	82 (3.6%)	2 (2.9%)	4 (1.4%)	18 (2.2%)	58 (5.4%)	
Secondary	1,333 (59%)	36 (51%)	140 (47%)	478 (58%)	679 (63%)	
Tertiary	849 (38%)	32 (46%)	152 (51%)	326 (40%)	339 (32%)	
Smoking						< 0.001***
Never	1,095 (51%)	51 (78%)	174 (61%)	425 (53%)	445 (44%)	
Former, > 6 months	516 (24%)	9 (14%)	63 (22%)	202 (25%)	242 (24%)	
Former, < 6 months	52 (2.4%)	1 (1.5%)	8 (2.8%)	15 (1.9%)	28 (2.7%)	
Current	499 (23%)	4 (6.2%)	38 (13%)	153 (19%)	304 (30%)	
Physical activity						0.601
Inactive	448 (21%)	11 (17%)	63 (22%)	160 (20%)	214 (21%)	
Lightly active	910 (42%)	25 (39%)	105 (37%)	345 (44%)	435 (43%)	
Active	723 (34%)	24 (38%)	105 (37%)	265 (33%)	329 (32%)	
Very active	74 (3.4%)	4 (6.3%)	9 (3.2%)	23 (2.9%)	38 (3.7%)	
Alcohol consumption (g/day)	11.82 (19.24)	8.49 (16.04)	13.06 (21.69)	10.85 (17.77)	12.44 (19.77)	0.077
Sleep quality						0.721
Satisfactory	1,239 (59%)	42 (67%)	158 (57%)	472 (61%)	567 (58%)	
Somewhat unsatisfactory	687 (33%)	15 (24%)	94 (34%)	248 (32%)	330 (34%)	
Significantly unsatisfactory	158 (7.5%)	5 (7.9%)	22 (7.9%)	52 (6.7%)	79 (8.0%)	
Totally unsatisfactory	19 (0.9%)	1 (1.6%)	4 (1.4%)	6 (0.8%)	8 (0.8%)	
*Anthropometrics*						
BMI (kg/m^2^)	26.92 (4.83)	27.17 (5.03)	26.87 (5.15)	27.09 (5.01)	26.79 (4.59)	0.587
BMI category (WHO)						0.099
Underweight	13 (0.6%)	1 (1.5%)	4 (1.4%)	5 (0.6%)	3 (0.3%)	
Normal weight	842 (37%)	20 (30%)	111 (38%)	296 (36%)	415 (39%)	
Overweight	909 (40%)	32 (48%)	108 (37%)	325 (40%)	444 (42%)	
Obese	482 (21%)	14 (21%)	71 (24%)	191 (23%)	206 (19%)	
Waist circumference (cm)	92.07 (13.59)	92.52 (14.69)	92.24 (14.37)	91.96 (13.67)	92.09 (13.27)	0.987
Hip circumference (cm)	99.96 (9.57)	100.75 (10.73)	100.86 (10.24)	100.09 (9.79)	99.56 (9.12)	0.242
Waist–hip ratio	0.92 (0.08)	0.91 (0.08)	0.91 (0.09)	0.92 (0.08)	0.92 (0.09)	0.206
*Body composition*						
Body fat percentage (%)	28.54 (9.21)	29.67 (9.74)	30.40 (9.10)	29.64 (9.36)	27.14 (8.89)	< 0.001***
Body fat mass (kg)	23.13 (10.77)	24.40 (11.93)	24.51 (11.16)	23.80 (11.13)	22.17 (10.23)	0.002**
Skeletal muscle mass (kg)	31.42 (7.08)	30.88 (7.27)	30.17 (7.03)	30.56 (6.91)	32.46 (7.06)	< 0.001***
Visceral fat area (cm²)	105.23 (41.37)	108.50 (46.22)	108.57 (43.28)	107.35 (42.37)	102.52 (39.63)	0.048*

Values are mean (SD) or n (%). *P*-values from Welch’s ANOVA and χ^2^ test; Monte Carlo simulation used when expected counts < 5. Significance levels: ***p < 0.001; **p < 0.01; *p < 0.05.

WHO, World Health Organization.

Lifestyle and sociodemographic factors varied across coffee consumption groups. Educational attainment differed by intake (*p* < 0.001), with tertiary education most common in low consumers (51%) and least common in high consumers (32%). High coffee consumers were also more likely to be current smokers compared with lower consumers (*p* < 0.001). Anthropometric measures, including BMI, waist circumference, and WHR, were similar between coffee categories.

Body composition showed significant differences across coffee consumption groups. Higher coffee intake groups showed lower body fat percentage (*p* < 0.001), lower body fat mass (*p* = 0.002), lower visceral fat area (*p* = 0.048), and higher skeletal muscle mass (*p* < 0.001).

A full overview of all baseline characteristics, including the number of participants with missing data for each variable and *p*-values for coffee by sex interaction terms for continuous and binary variables, is provided in Supplementary Table S1. Sensitivity analyses excluding participants with extreme BMI values (< 18.5 or > 40 kg/m²) showed similar anthropometric and body composition patterns across coffee consumption groups (Supplementary Table S2).

### Correlations between coffee consumption and circulating metabolites

**Fig. 2:**
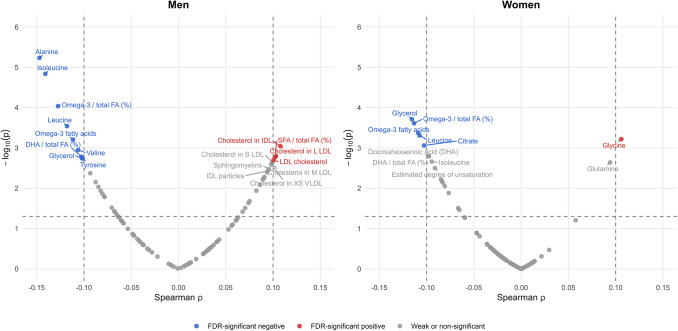
Sex-stratified Spearman correlations between coffee intake and circulating metabolites

Spearman correlation analyses were performed to assess unadjusted correlations between habitual coffee intake and serum metabolites quantified by NMR spectroscopy, stratified by sex. The sex-stratified correlation coefficients and FDR-corrected *p*-values are reported in Supplementary Table S3 and visualized as combined volcano plots in Fig. 2. Although correlation magnitudes were generally small (|ρ| < 0.15), several correlations remained significant after FDR correction.

In men, coffee consumption showed a broader pattern of significant correlations than in women. Higher coffee intake was inversely correlated with several amino acids, including the branched-chain amino acids (BCAAs) isoleucine, leucine, and valine, as well as alanine, omega-3 fatty acids, and their relative proportions. Conversely, positive correlations were observed with several cholesterol-related measures, including LDL- and IDL-related cholesterol measures and the proportion of saturated fatty acids.

Volcano plots show sex-stratified Spearman rank correlations (ρ) between habitual coffee consumption (cups/day) and circulating serum metabolites quantified by NMR spectroscopy. The x-axis represents correlation coefficients (ρ), and the y-axis represents −log10 nominal *p*-values. Horizontal dashed lines indicate nominal *p* = 0.05, and vertical dashed lines indicate |ρ| = 0.10. Coloured points indicate correlations meeting both thresholds: FDR-adjusted q < 0.05 and |ρ| ≥ 0.10.

In women, the correlation pattern was more limited. Coffee intake was inversely correlated with metabolites involved in lipid and energy metabolism, including glycerol, citrate, leucine, and omega-3 fatty acids, and positively correlated with glycine.

Overall, coffee consumption showed sex-specific metabolic correlation patterns, with a broader pattern observed in men.

### Correlations between coffee consumption and cardiometabolic and hormonal markers

**Fig. 3:**
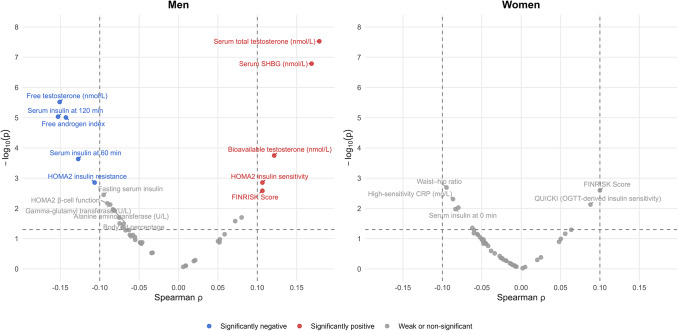
Sex-stratified correlations between coffee intake and cardiometabolic and hormonal markers

Correlations between coffee intake and cardiometabolic risk markers are presented in Fig. 3 and reported in Supplementary Table S4. Most correlations were small (|ρ| < 0.2), although several were statistically significant.

In men, higher coffee intake was inversely correlated with fasting insulin, OGTT insulin responses (60 and 120 min), and HOMA2 indices of insulin resistance and β-cell function, and positively correlated with SHBG, total and bioavailable testosterone, HOMA2 insulin sensitivity, and the FINRISK score.

Volcano plots show sex-stratified Spearman rank correlations (ρ) between habitual coffee consumption (cups/day) and cardiometabolic and hormonal markers. The x-axis represents correlation coefficients (ρ), and the y-axis represents −log10(*p*-values). Horizontal dashed lines indicate *p* = 0.05, and vertical dashed lines indicate |ρ| = 0.10. Coloured points indicate correlations meeting both thresholds: *p* < 0.05 and |ρ| ≥ 0.10.

In women, the correlation pattern was less pronounced, however modest inverse correlations were observed with waist-to-hip ratio, hsCRP, and fasting insulin, whereas modest positive correlations were observed with the FINRISK score and QUICKI-derived insulin sensitivity.

Overall, habitual coffee intake showed sex-specific correlation patterns with cardiometabolic and hormonal markers, with a broader and more consistent pattern observed in men.

### Associations between coffee intake and hormonal markers

*Serum total testosterone across coffee consumption groups*

**Fig. 4:**
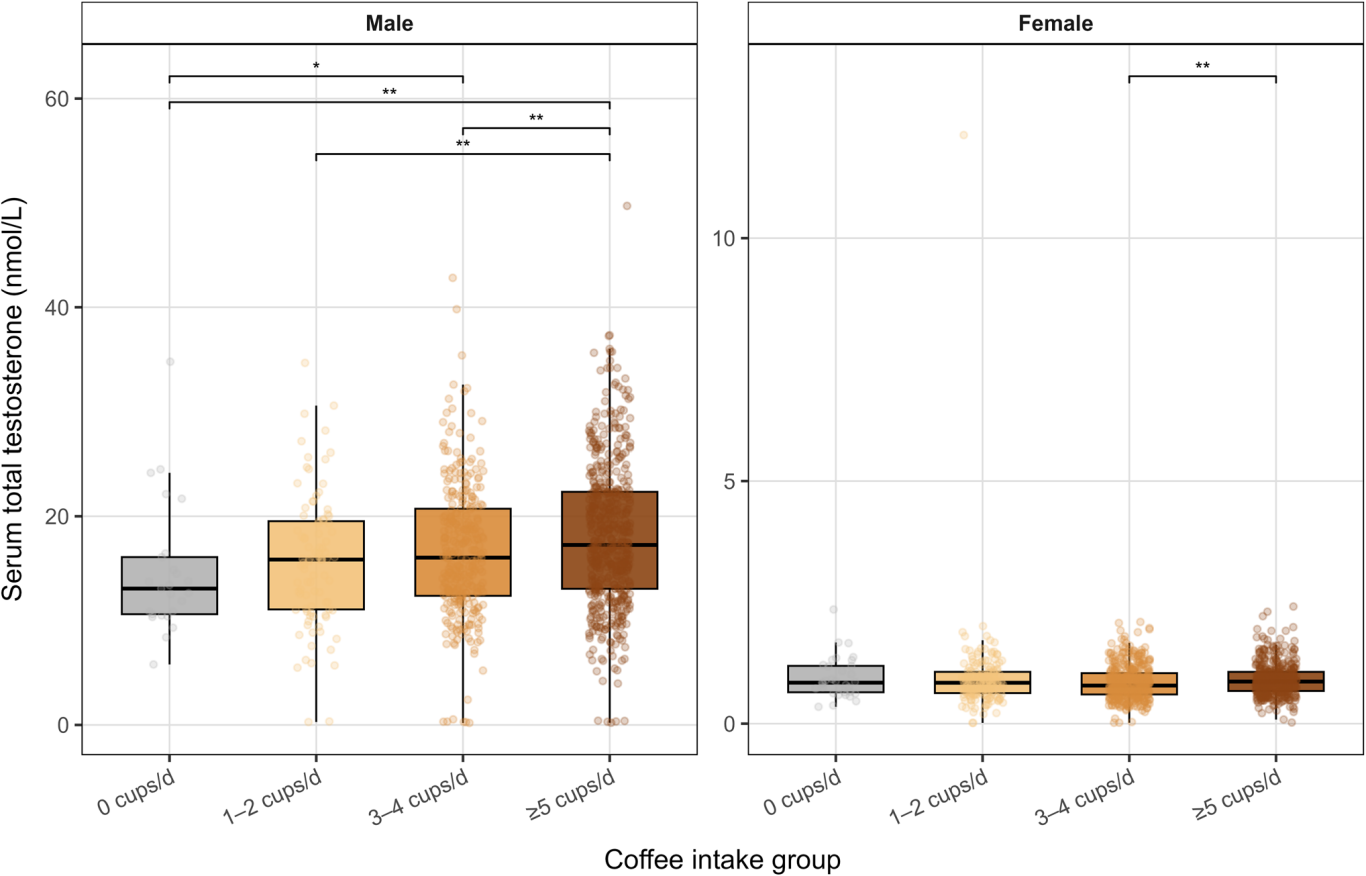
Serum total testosterone by coffee intake group, stratified by sex

To visualize the sex-specific differences in serum total testosterone across coffee consumption groups, testosterone concentrations were compared between habitual coffee intake categories using pairwise Wilcoxon rank-sum tests (Fig. 4).

Boxplots show serum total testosterone concentrations across habitual coffee consumption groups, stratified by sex. Boxes indicate the interquartile range, with medians shown as horizontal lines; whiskers extend to 1.5× the interquartile range, and points represent individual participants, excluding outliers. Between-group differences were assessed within each sex using pairwise Wilcoxon rank-sum tests. Significant comparisons are annotated as *p* < 0.05*, *p* < 0.01**.

In men, serum total testosterone tended to increase across higher coffee consumption groups. Testosterone levels were significantly higher in moderate (*p* < 0.05) and high consumers (*p* < 0.01) compared with non-consumers. Differences were also observed between coffee intake groups, with high consumers showing higher levels than both low and moderate consumers (both *p* < 0.01).

In women, testosterone concentrations were low overall and showed no consistent differences across intake groups, except for a higher value in high compared with moderate consumers (*p* < 0.01).

*Multivariable associations between coffee intake and hormonal markers*

**Fig. 5:**
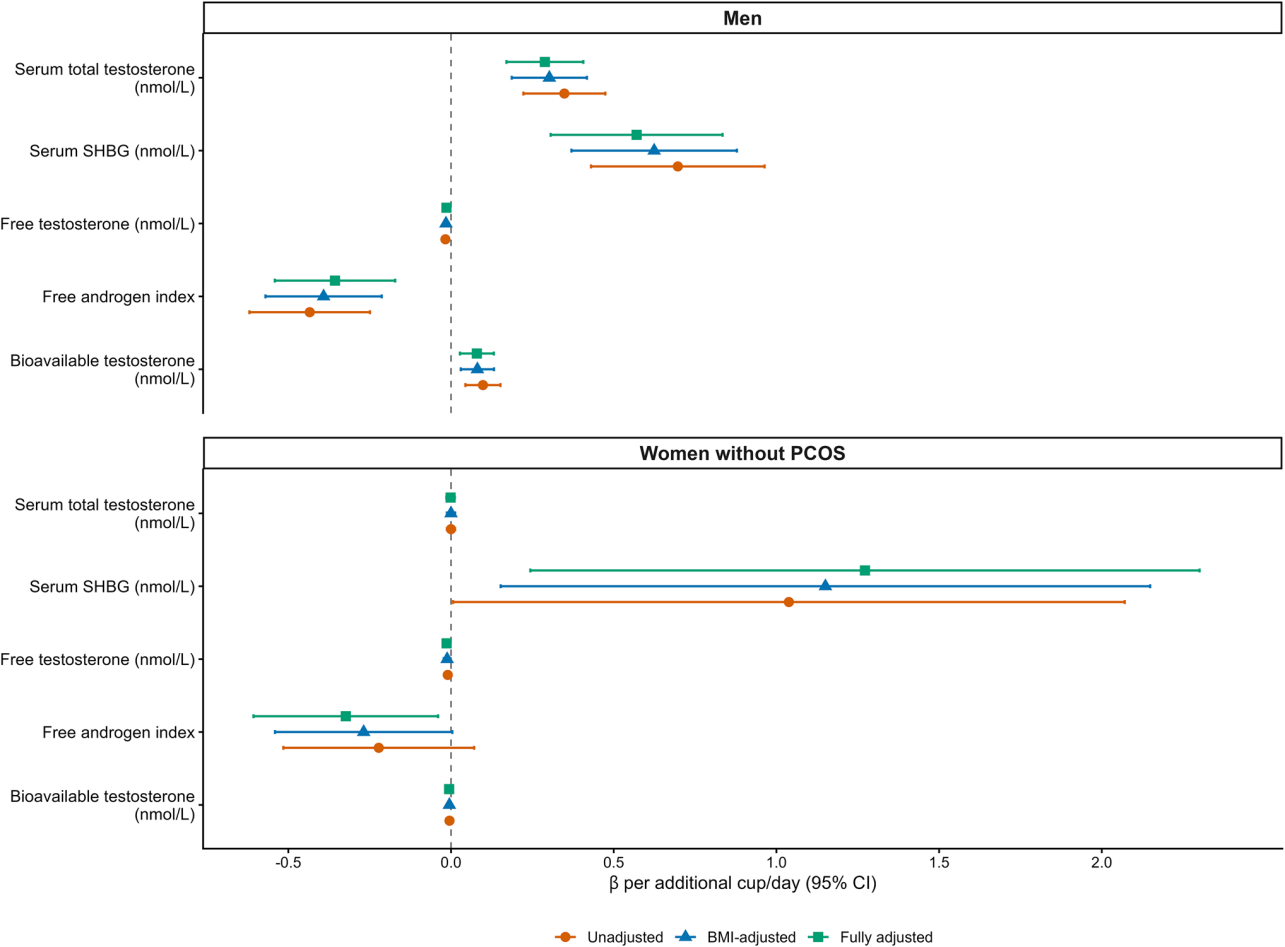
Multivariable associations between coffee intake and hormonal markers, stratified by sex

To further evaluate these associations while accounting for potential confounders, multivariable linear regression analyses were performed for key hormonal markers, including total, bioavailable, and free testosterone, SHBG, and the FAI. The sex-stratified results are shown in Fig. 5, while full model details, including sample sizes, β-estimates, 95% confidence intervals, and *p*-values, are provided in Supplementary Table S6. In the fully adjusted models, analyses included approximately 896 men and 937 women, with female analyses restricted to women with available PCOS data who reported no PCOS diagnosis. Results are expressed as β-estimates per additional cup of coffee per day. Exploratory sex-stratified generalized additive models using a smooth term for coffee intake did not show meaningful improvement over the corresponding linear models for most hormonal outcomes, supporting the use of linear regression as the primary approach (Supplementary Table S5).

Forest plot showing β-estimates and 95% confidence intervals from sex-stratified linear regression models assessing the association between habitual coffee intake (cups/day) and circulating hormonal markers. Estimates represent the change in each outcome per additional cup of coffee per day. Models are shown as unadjusted, BMI-adjusted, and fully adjusted. The fully adjusted model included BMI, educational attainment, smoking status, physical activity, and alcohol consumption. Female analyses were restricted to women with available PCOS data and no reported PCOS diagnosis. Full numerical results, including sample sizes and *p*-values, are provided in Supplementary Table S6.

In men, higher coffee intake was positively associated with serum total testosterone (β = +0.29 nmol/L per cup/day, *p* < 0.001), bioavailable testosterone (β = +0.08 nmol/L per cup/day, *p* = 0.003), and SHBG (β = +0.57 nmol/L per cup/day, *p* < 0.001) in the fully adjusted model. Inverse associations were observed with the FAI (β = −0.36 per cup/day, *p* < 0.001) and free testosterone (β = −0.01 nmol/L per cup/day, *p* < 0.001). These associations were already evident in the unadjusted model and remained statistically significant after adjustment for BMI and lifestyle factors.

In women, coffee intake was positively associated with SHBG in the fully adjusted model (β = +1.27 nmol/L per cup/day, *p* = 0.015). Inverse associations were observed with the FAI (β = −0.32 per cup/day, *p* = 0.026) and free testosterone (β = −0.01 nmol/L per cup/day, *p* = 0.015). No significant associations were observed with total or bioavailable testosterone.

## Discussion

Using cross-sectional data from 46-year-old adults in the NFBC1966, we examined how habitual coffee intake relates to circulating metabolites, cardiometabolic risk factors and hormonal markers.

We observed differences in body composition across coffee consumption groups. While BMI, waist circumference, hip circumference, and waist–hip ratio were similar between groups, individuals with higher coffee intake had lower BIA-derived body fat percentage, lower absolute fat mass, lower visceral fat area, and higher skeletal muscle mass, showing dose-related trends. Together, these findings suggest that regular coffee drinkers exhibited a lower adiposity profile despite similar BMI. However, these findings should be interpreted cautiously, as BIA-derived body composition estimates are indirect and rely on assumptions related to body water and tissue conductivity [29].

The lower adiposity profile observed among higher coffee consumers is consistent with a growing body of experimental, genetic, and epidemiological evidence linking coffee and caffeine intake to adiposity-related outcomes. Mendelian randomization analyses indicate that genetically predicted higher plasma caffeine concentrations are associated with lower BMI and whole-body fat mass, and reduced T2D risk [30].

Mechanistically, caffeine acts as an adenosine-receptor antagonist, activating the sympathetic nervous system and increasing resting metabolic rate, energy expenditure, and thermogenic lipolysis [31, 32]. These acute effects translate into enhanced substrate utilization, as meta-analyses show that pre-exercise caffeine intake enhances fat oxidation and oxygen uptake during aerobic exercise in both fasting and fed states [33, 34]. Over time, these mechanisms may contribute to weight regulation through increased lipid turnover, higher metabolic rate, and reduced absorption of short-chain fatty acids [12, 30, 35].

Consistent with these mechanisms, short-term randomized trials show that regular coffee consumption can reduce body fat and energy intake while modulating satiety hormones, such as serotonin and ghrelin [36]. A 24-week randomized placebo-controlled trial further demonstrated a modest but significant reduction in fat mass with daily caffeinated coffee intake, even in the absence of changes in insulin sensitivity [37]. In parallel, a pilot randomized controlled trial (RCT) showed that dietary fiber from spent coffee grounds increased satiety and reduced *ad libitum* energy intake, suggesting a potential role in appetite regulation [38].

Regarding blood pressure, diastolic BP did not differ across coffee consumption groups, whereas systolic BP showed a small overall difference across groups. Mean systolic BP was highest among high coffee consumers, but the absolute differences were modest, “ranging from ~ 1.6 mmHg higher than moderate consumers to ~ 3.6 mmHg higher than non-consumers. Because BP was measured after a 12-hour overnight fast and abstention from coffee, this small difference is unlikely to reflect acute caffeine effects. Although this contrasts with prospective studies and dose–response meta-analyses reporting a modestly lower hypertension risk among habitual coffee drinkers [39–42]. The magnitude of the difference was substantially lower than the transient pressor response reported after acute caffeine intake (200–300 mg), which can increase systolic and diastolic BP by approximately 8 and 6 mmHg, respectively [12, 43].

Spearman correlation analyses of circulating metabolites revealed a broader pattern of correlations in men than in women. In men, higher coffee intake correlated inversely with several circulating amino acids, including alanine, isoleucine, leucine, valine and tyrosine, as well as omega-3 fatty acids and glycerol, and correlated positively with LDL- and IDL-related lipid fractions and a higher proportion of SFA. In women, the metabolic correlation pattern was narrower, with inverse correlations primarily for glycerol, omega-3 fatty acids, leucine and citrate, and positive correlations for glutamine and glycine. These findings extend the existing metabolomics literature by showing inverse correlations between coffee intake and several circulating amino acids, including the BCAAs.

The inverse correlations between coffee intake and circulating BCAAs in both men and women are particularly noteworthy. Although BCAAs are essential amino acids involved in key metabolic signalling pathways, chronically elevated levels are strongly associated with insulin resistance, T2D, cardiovascular disease, fatty liver disease, and increased hypertension risk [44–49]. These adverse associations appear to reflect endogenous BCAA accumulation and impaired metabolic regulation rather than dietary intake per se [50]. Supporting this interpretation, elevated BCAAs predict future T2D years before clinical onset [51]. In line with this, reductions in circulating BCAAs during weight loss have been linked to improvements in insulin sensitivity and skeletal-muscle lipid oxidation [52–54].

The positive correlations observed for LDL- and IDL-related fractions may be biologically plausible, as coffee diterpenes such as cafestol and kahweol can increase apolipoprotein B–containing lipoproteins through inhibition of bile acid synthesis [55–58]. However, this interpretation should be cautious, because diterpene-related LDL increases are mainly linked to unfiltered coffee, whereas coffee consumption in Finland is predominantly filtered, which substantially reduces diterpene exposure. This may partly explain why evidence on coffee intake and LDL-related outcomes remains mixed [12, 59, 60]. In men, we also observed a higher proportion of SFA and inverse correlations with omega-3 fatty acids and DHA. These findings contrast with limited intervention evidence reporting no change in absolute SFA and increases in DHA and the omega-3 index after dark-roasted coffee [61]. Given that Finns typically consume light-roasted coffee, this discrepancy may reflect differences in roast-dependent bioactive profiles, as well as sex-related variation in coffee bioactive metabolism.

Spearman correlation analyses of cardiometabolic and hormonal markers also showed a clear sex-specific pattern. In men, higher coffee intake was correlated with lower fasting insulin, lower insulin concentrations during the OGTT, reduced HOMA2-derived insulin resistance and β-cell function, and higher insulin sensitivity. These correlations suggest that habitual coffee consumption may be linked to a more favourable glucose–insulin profile in men, which is broadly consistent with evidence from observational studies and longer-term trials showing improved glycaemic regulation and lower T2D risk among regular coffee drinkers [62]. While acute studies indicate that coffee and caffeine transiently impair glucose tolerance, chronic coffee intake has been associated with enhanced insulin sensitivity over time, although previous reviews have not identified consistent sex-specific effects [63, 64]. In women, the correlations were fewer and weaker, centred on adiposity and inflammation, including an inverse correlation with waist–hip ratio, together with a positive association with the FINRISK score. A similar marginally positive association with FINRISK score was also observed in men. Given that this score is a composite 10-year cardiovascular risk estimate driven by smoking, blood pressure, lipids, diabetes and family history [27], this likely reflects correlated lifestyle patterns rather than a direct adverse effect of coffee itself.

Importantly, hormonal markers revealed pronounced sex specificity, with men showing positive correlations between habitual coffee intake and total and bioavailable testosterone and SHBG, and inverse correlations with free testosterone and the FAI, whereas these correlations were not observed in women. Given their magnitude and clear sex specificity, these hormonal findings were examined in more detail using regression models adjusted for covariates. Focusing on these hormonal associations, multivariable regression models demonstrated a clear graded pattern in men. Total testosterone increased progressively across coffee consumption categories, with significantly higher concentrations in moderate and high consumers compared with non-consumers. These dose-related differences were supported by the adjusted regression models, in which higher coffee intake was positively associated with total and bioavailable testosterone and with SHBG, and inversely associated with free testosterone and FAI. The consistency of these associations across all adjustment models suggests that the observed hormonal pattern is not fully explained by differences in adiposity or lifestyle factors. In women, testosterone concentrations were considerably lower overall and showed no clear pattern across intake categories. In the adjusted analyses (restricted to women without PCOS), coffee intake was positively associated with SHBG and inversely associated with free testosterone and the FAI, but no associations were observed with total or bioavailable testosterone.

Our results indicate that coffee consumption is associated with a broader androgen-related profile in men, characterized by higher total and bioavailable testosterone and higher SHBG, together with lower free testosterone, whereas in women the associations are limited to SHBG and free hormone fractions. The clinical relevance of this pattern merits consideration. In a large nationally representative sample of US men (NHANES III), lower concentrations of total testosterone and SHBG were independently associated with a two-fold or greater likelihood of MetS after adjustment for age, adiposity, smoking, physical activity, LDL cholesterol, CRP, and insulin resistance. Notably, calculated free testosterone and bioavailable testosterone were not independently associated with MetS after full adjustment, underscoring that total testosterone and SHBG may be more metabolically informative fractions [65]. Moreover, SHBG has emerged across multiple large prospective studies and meta-analyses as an independent predictor of T2D risk in both men and women, with low SHBG further associated with coronary artery disease in postmenopausal women and peripheral artery disease in older individuals, independent of established cardiovascular risk factors [66].

The concurrent coffee-associated increase in total testosterone and SHBG, alongside a reduction in the free androgen index, therefore, maps onto a hormonally favourable profile that may be relevant to metabolic and cardiometabolic health at the population level. This pattern aligns in part with findings from animal models, where caffeine or chlorogenic-acid–rich coffee has been shown to increase testosterone or enhance androgen signalling in models of diabetes and heat-induced testicular stress, as well as following chronic low-dose caffeine exposure, possibly through improved steroidogenesis, reduced oxidative stress, and preserved testicular structure [67–70]. While not directly comparable to habitual human intake, these findings indicate that coffee compounds could interact with androgen-regulatory pathways.

Human interventional studies, however, suggest mainly transient effects. Acute caffeine intake, particularly in exercise settings, can substantially raise circulating testosterone. Controlled trials show that high doses of caffeine (up to 800 mg) can amplify exercise-induced testosterone increases by more than 20%, accompanied by parallel rises in cortisol [71]. Similar acute responses have been observed with caffeinated coffee, which produced the highest post-exercise testosterone in trained athletes and exceeded the effects of pure caffeine [72]. Notably, the same study also reported elevated testosterone levels after decaffeinated coffee, suggesting that caffeine itself may not fully account for the acute hormonal response and that other coffee-derived bioactives could contribute. In contrast, evidence for longer-term effects is limited: one RCT reported a modest increase in total testosterone at week 4, which did not persist at week 8, and no consistent changes in SHBG [20]. However, because testosterone shows substantial biological and analytical variability [73, 74], these modest cross-sectional associations are best interpreted as population-level endocrine patterns rather than direct evidence of clinically meaningful hormonal changes within individuals.

In observational data, results from adult populations show a more consistent pattern for SHBG than for testosterone, with notable differences between men and women. In men, both the Tromsø Study and National Health and Nutrition Examination Survey (NHANES) III initially reported higher total testosterone with greater coffee consumption; however, in NHANES III this association became non-significant after adjusting for factors such as adiposity, smoking, alcohol use, and physical activity [21, 75]. SHBG, by contrast, remained positively associated with coffee intake in both cohorts. Additional analyses of NHANES data from 1999 to 2004 and 2011–2012 found no linear relationship between caffeine intake and testosterone, and the observed J-shaped patterns were inconsistent across subgroups [76]. More recent NHANES 2013–2014 metabolite studies further extend this picture, showing inverse associations between caffeine metabolites and testosterone in adult men, as well as in male children and adolescents, suggesting a broadly similar pattern across age groups [18, 77].

Beyond testosterone and SHBG, observational evidence does not indicate major adverse reproductive effects of coffee. The Health Professionals Follow-up Study reported no association between regular coffee intake and erectile dysfunction [78], and a systematic review of 28 studies found no consistent links between coffee or caffeine intake and semen quality, with negative findings more commonly attributed to soft drinks rather than coffee [79].

In women, two large cohort studies provide relevant insight. In the Nurses’ Health Study, higher caffeine and coffee intake were linked to lower luteal oestradiol in premenopausal women and higher SHBG in postmenopausal women, with no associations with total or free testosterone [80]. In the Women’s Health Study, caffeinated coffee was similarly associated with higher SHBG, while sex hormones remained unchanged; this SHBG increase partly mediated the lower T2D risk among high coffee consumers [81]. Overall, these findings indicate that in women, coffee intake relates mainly to higher SHBG and modest oestradiol shifts, rather than changes in testosterone, consistent with the sex-specific patterns observed in our cohort.

### Strengths and limitations

A major strength of this study is the use of NFBC1966, a large population-based birth cohort comprising nearly all births in Northern Finland in 1966, with extensive prospective data collection across the life course and a high coffee consumption. The homogeneous age and ethnic background of participants reduces potential confounding from age- and ancestry-related variation in hormonal and metabolic profiles, strengthening the internal validity of the observed associations. The 46-year follow-up provides clinical examinations, standardized fasting protocols, and detailed lifestyle and health assessments, enabling robust evaluation of metabolic and hormonal profiles. The availability of comprehensive biomarker data, including NMR metabolomics and androgen-related hormones, together with a large sample size and sex-stratified analyses, increases analytic power and allows the detection of sex-specific associations. Adjustment for sociodemographic, lifestyle, and adiposity-related factors further strengthens internal validity.

Several limitations should be noted. The cross-sectional design precludes causal inference, and reverse causation cannot be excluded. Residual confounding from unmeasured factors such as dietary patterns, stress, and menstrual cycle phase in women also cannot be ruled out. Hormonal markers were assessed at a single time point, and given the well-documented intra-individual variability of testosterone [73], the observed associations reflect population-level patterns rather than individually detectable clinical changes. Coffee intake was self-reported, and detailed information on cup size, brewing method, bean type, and additions was unavailable, which may introduce exposure misclassification and limits insight into which specific bioactive compounds drive the observed effects. Finally, while the analytical sample was broadly representative of the clinically examined cohort, the relatively small number of non-consumers and the homogeneous Finnish population may limit the generalizability of findings to populations with different coffee cultures and consumption patterns.

## Conclusion

This study provides population-level evidence that habitual coffee consumption is associated with a sex-differentiated metabolic and hormonal profile in middle-aged adults. Higher coffee intake groups showed lower total and visceral fat despite similar BMI, together with inverse correlations with circulating BCAAs in both men and women, a potentially novel finding that may be relevant to insulin resistance and future cardiometabolic risk.

The most pronounced sex-specific finding was the androgen-related hormonal profile in men: higher coffee intake was independently associated with higher total testosterone, bioavailable testosterone, and SHBG, alongside a lower FAI. Both total testosterone and SHBG are established independent predictors of MetS and T2D risk in men, making the observed pattern hormonally coherent with the cardiometabolic associations. In women, hormonal associations were more limited and centred on higher SHBG and lower FAI, consistent with the SHBG-mediated pathway previously shown to partly explain coffee’s protective association with T2D in women. Together, our findings indicate that habitual coffee consumption is associated with a broader endocrine–metabolic signature that differs by sex.

Future longitudinal and interventional studies are needed to clarify whether these associations reflect direct biological effects of coffee constituents on endocrine and metabolic pathways, and to identify the bioactive compounds involved.

## Supplementary Information

Below is the link to the electronic supplementary material.

Supplementary Material 1

## Data Availability

The data used in this study are derived from the NFBC1966 and are available from the University of Oulu, Infrastructure for Population Studies. Access to the data is subject to approval and can be requested for research purposes through the cohort’s electronic material request portal. All data handling complies with the EU General Data Protection Regulation (679/2016) and the Finnish Data Protection Act. Use of personal data is based on the written informed consent provided by participants at their most recent follow-up. Further details are available from the NFBC project center (NFBCprojectcenter@oulu.fi) and on the cohort website (www.oulu.fi/nfbc).
