# Supplementary material for: Associations of habitual coffee intake with testosterone and cardiometabolic markers: the Northern Finland birth cohort 1966 study
Source: Eur J Nutr. 2026 Jul 16;65(5):215. doi: 10.1007/s00394-026-04038-z (PMC13375829; doi:10.1007/s00394-026-04038-z)
Supplement: Supplementary file 1 — Supplementary Material 1 [file 394_2026_4038_MOESM1_ESM.pdf]

Supplementary Material

European Journal of Nutrition – Luca Verroest et al., Luca.Verroest@Oulu.fi

Associations of Habitual Coffee Intake with Testosterone and Cardiometabolic Markers: the Northern Finland Birth Cohort 1966 Study

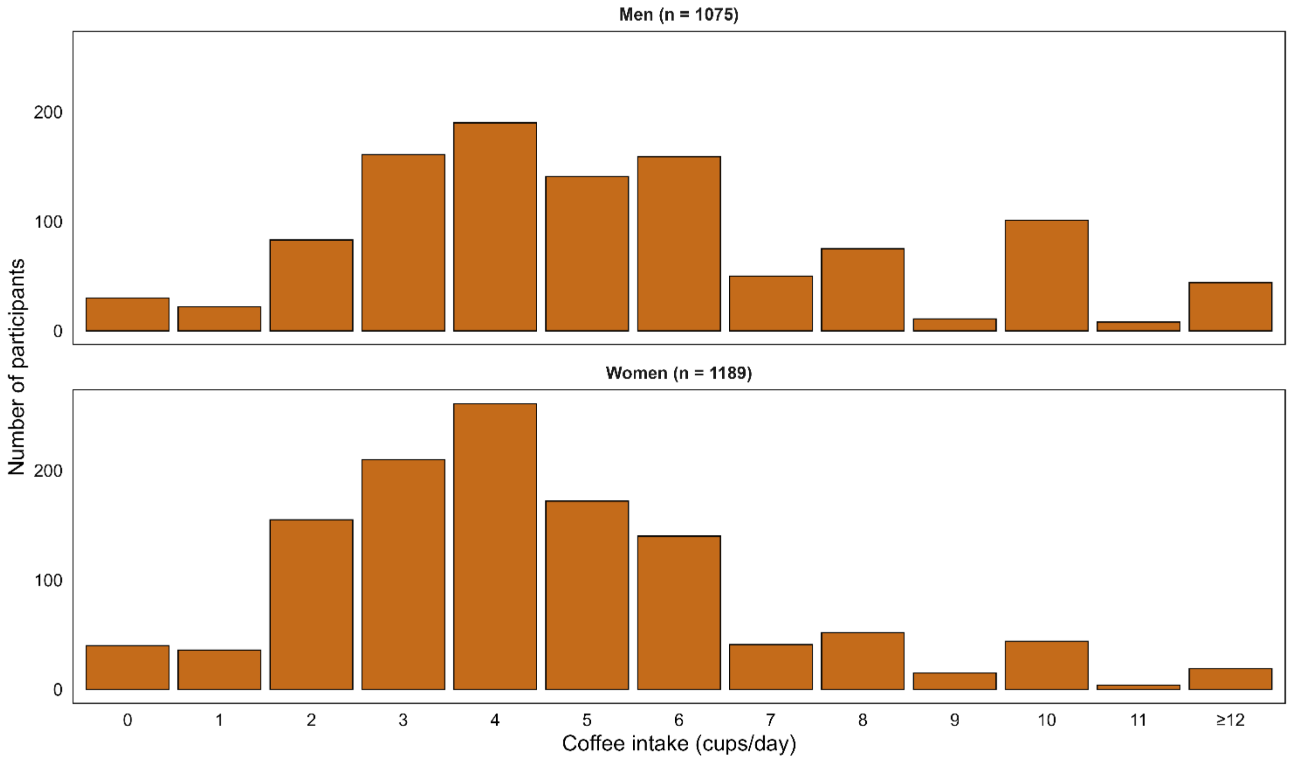

Supplementary Fig. S1 Distribution of habitual coffee intake (cups/day) among men and women in the NFBC1966 at age 46 years

Supplementary Material

European Journal of Nutrition – Luca Verroest et al., Luca.Verroest@Oulu.fi

Supplementary Table S1 Extended participant characteristics by categories of habitual coffee consumption

Measure	Missing (n)	Overall	Non-consumers	1–2 cups/d	3–4 cups/d	≥5 cups/d	*p*-value	*p*-interaction
		N = 2,264	N = 70	N = 296	N = 822	N = 1,076		
**Sociodemographics & Lifestyle**								
Sex	0						**<0.001*****	
Men		1,075 (47%)	30 (43%)	105 (35%)	351 (43%)	589 (55%)		
Women		1,189 (53%)	40 (57%)	191 (65%)	471 (57%)	487 (45%)		
Education level	0						**<0.001*****	
Basic or less		82 (3.6%)	2 (2.9%)	4 (1.4%)	18 (2.2%)	58 (5.4%)		
Secondary		1,333 (59%)	36 (51%)	140 (47%)	478 (58%)	679 (63%)		
Tertiary		849 (38%)	32 (46%)	152 (51%)	326 (40%)	339 (32%)		
Smoking	102						**<0.001*****	
Never		1,095 (51%)	51 (78%)	174 (61%)	425 (53%)	445 (44%)		
Former, > 6 months		516 (24%)	9 (14%)	63 (22%)	202 (25%)	242 (24%)		
Former, < 6 months		52 (2.4%)	1 (1.5%)	8 (2.8%)	15 (1.9%)	28 (2.7%)		
Current		499 (23%)	4 (6.2%)	38 (13%)	153 (19%)	304 (30%)		
Physical activity	109						0.601	
Inactive		448 (21%)	11 (17%)	63 (22%)	160 (20%)	214 (21%)		
Lightly active		910 (42%)	25 (39%)	105 (37%)	345 (44%)	435 (43%)		
Active		723 (34%)	24 (38%)	105 (37%)	265 (33%)	329 (32%)		
Very active		74 (3.4%)	4 (6.3%)	9 (3.2%)	23 (2.9%)	38 (3.7%)		
Alcohol consumption (g/day)	96	11.82 (19.24)	8.49 (16.04)	13.06 (21.69)	10.85 (17.77)	12.44 (19.77)	0.077	0.799
Sleep quality	161						0.721	
Satisfactory		1,239 (59%)	42 (67%)	158 (57%)	472 (61%)	567 (58%)		
Somewhat unsatisfactory		687 (33%)	15 (24%)	94 (34%)	248 (32%)	330 (34%)		
Significantly unsatisfactory		158 (7.5%)	5 (7.9%)	22 (7.9%)	52 (6.7%)	79 (8.0%)		
Totally unsatisfactory		19 (0.9%)	1 (1.6%)	4 (1.4%)	6 (0.8%)	8 (0.8%)		
**Anthropometrics**								
BMI (kg/m^2)	18	26.92 (4.83)	27.17 (5.03)	26.87 (5.15)	27.09 (5.01)	26.79 (4.59)	0.587	0.375
BMI category (WHO)	18						0.099	
Underweight		13 (0.6%)	1 (1.5%)	4 (1.4%)	5 (0.6%)	3 (0.3%)		

Normal weight		842 (37%)	20 (30%)	111 (38%)	296 (36%)	415 (39%)		
Overweight		909 (40%)	32 (48%)	108 (37%)	325 (40%)	444 (42%)		
Obese		482 (21%)	14 (21%)	71 (24%)	191 (23%)	206 (19%)		
Waist circumference (cm)	261	92.07 (13.59)	92.52 (14.69)	92.24 (14.37)	91.96 (13.67)	92.09 (13.27)	0.987	0.817
Hip circumference (cm)	262	99.96 (9.57)	100.75 (10.73)	100.86 (10.24)	100.09 (9.79)	99.56 (9.12)	0.242	0.642
Waist–hip ratio	262	0.92 (0.08)	0.91 (0.08)	0.91 (0.09)	0.92 (0.08)	0.92 (0.09)	0.206	0.783
**Body composition**								
Body fat percentage	273	28.54 (9.21)	29.67 (9.74)	30.40 (9.10)	29.64 (9.36)	27.14 (8.89)	**<0.001*** **	0.541
Body fat mass (kg)	273	23.13 (10.77)	24.40 (11.93)	24.51 (11.16)	23.80 (11.13)	22.17 (10.23)	**0.002****	0.598
Skeletal muscle mass (kg)	273	31.42 (7.08)	30.88 (7.27)	30.17 (7.03)	30.56 (6.91)	32.46 (7.06)	**<0.001*** **	0.555
Visceral fat area (cm²)	273	105.23 (41.37)	108.50 (46.22)	108.57 (43.28)	107.35 (42.37)	102.52 (39.63)	**0.048***	0.647
**Blood pressure (mmHg)**								
Mean systolic BP	255	125.35 (15.62)	122.80 (16.69)	123.62 (14.91)	124.80 (15.78)	126.38 (15.57)	**0.020***	0.425
Mean diastolic BP	255	84.84 (10.66)	83.16 (11.91)	84.50 (10.75)	84.69 (10.58)	85.15 (10.63)	0.502	0.319
**Fasting serum lipids (mmol/L)**								
Total cholesterol	251	5.34 (0.94)	5.27 (0.69)	5.32 (0.94)	5.30 (0.90)	5.39 (0.97)	0.180	0.373
HDL cholesterol	249	1.56 (0.41)	1.51 (0.39)	1.57 (0.42)	1.56 (0.40)	1.55 (0.42)	0.734	0.559
LDL cholesterol	249	3.45 (0.94)	3.31 (0.61)	3.40 (0.98)	3.40 (0.92)	3.51 (0.97)	**0.025***	0.189
Triglycerides	249	1.27 (0.94)	1.52 (1.22)	1.39 (1.37)	1.28 (1.00)	1.22 (0.68)	**0.042***	0.077
**Fasting values**								
Fasting plasma glucose	286	5.54 (0.98)	5.47 (0.54)	5.70 (1.61)	5.53 (0.99)	5.50 (0.71)	0.195	0.539
Fasting serum insulin	275	9.86 (9.07)	12.78 (12.71)	11.02 (12.31)	9.77 (7.84)	9.42 (8.56)	0.064	0.710
**Medication**								
Lipid-modifying agents	88	109 (5.0%)	3 (4.5%)	14 (4.9%)	47 (5.9%)	45 (4.4%)	0.552	
Antihypertensive drugs	88	401 (18%)	15 (23%)	49 (17%)	157 (20%)	180 (18%)	0.490	
**Reproductive health**								
Polycystic ovary syndrome (PCOS)							0.224	
No		1,059 (89%)	31 (78%)	170 (89%)	427 (91%)	431 (89%)		
Yes		56 (4.7%)	3 (7.5%)	8 (4.2%)	21 (4.5%)	24 (4.9%)		
Missing		74 (6.2%)	6 (15%)	13 (6.8%)	23 (4.9%)	32 (6.6%)		

**OGTT (mmol/L)**								
Plasma glucose at 0 min	478	5.50 (0.76)	5.36 (0.52)	5.55 (0.79)	5.52 (0.92)	5.49 (0.61)	0.178	0.845
Plasma glucose at 30 min	510	8.12 (1.61)	8.06 (1.59)	8.28 (1.68)	8.04 (1.56)	8.14 (1.62)	0.276	0.365
Plasma glucose at 60 min	516	7.45 (2.46)	6.91 (2.42)	7.71 (2.59)	7.41 (2.43)	7.45 (2.44)	0.173	0.885
Plasma glucose at 120 min	505	5.82 (1.69)	5.63 (1.62)	6.01 (1.96)	5.86 (1.57)	5.74 (1.69)	0.169	0.980
Serum insulin at 0 min	476	9.74 (8.29)	10.40 (6.96)	10.98 (14.09)	9.95 (7.43)	9.19 (6.59)	0.057	0.377
Serum insulin at 30 min	510	73.79 (52.57)	84.51 (54.84)	74.99 (59.50)	74.17 (51.26)	72.49 (51.34)	0.454	**0.015***
Serum insulin at 60 min	509	86.66 (69.78)	94.13 (67.22)	99.88 (83.37)	85.34 (70.52)	83.54 (64.78)	**0.041***	**0.036***
Serum insulin at 120 min	501	62.42 (62.75)	71.28 (68.05)	75.59 (87.33)	64.52 (61.40)	56.60 (54.00)	**0.002****	0.217
**OGTT derived indices**								
Glucose AUC (0–180 min)	556	836.57 (191.68)	802.30 (183.93)	859.78 (210.50)	832.76 (186.83)	835.15 (190.02)	0.189	0.862
QUICKI (OGTT-derived insulin sensitivity)	500	0.35 (0.04)	0.35 (0.03)	0.35 (0.04)	0.35 (0.04)	0.35 (0.03)	0.270	0.424
**Insulin sensitivity/resistance**								
HOMA2 β-cell function	362	87.32 (31.34)	99.98 (37.06)	89.22 (35.61)	88.06 (31.51)	85.48 (29.34)	**0.013***	0.955
HOMA2 insulin sensitivity	362	107.63 (55.52)	94.16 (53.02)	105.56 (59.92)	107.31 (56.33)	109.26 (53.73)	0.195	0.959
HOMA2 insulin resistance	362	1.27 (0.86)	1.51 (1.05)	1.40 (1.01)	1.30 (0.91)	1.20 (0.75)	**0.004****	0.508
**Liver & kidney markers**								
Alanine aminotransferase (U/L)	249	31.88 (23.85)	28.53 (15.45)	32.47 (25.88)	31.66 (26.67)	32.09 (21.32)	0.402	0.204
Aspartate aminotransferase (U/L)	252	24.42 (15.72)	22.19 (6.76)	24.86 (20.67)	24.16 (16.93)	24.64 (13.44)	0.097	0.122
Gamma-glutamyl transferase (U/L)	251	36.06 (43.03)	32.97 (36.98)	43.32 (75.07)	36.63 (41.34)	33.82 (30.59)	0.118	**0.021***
Serum albumin (g/L)	249	45.16 (2.37)	45.36 (2.32)	44.88 (2.36)	45.02 (2.31)	45.33 (2.42)	**0.012***	0.481
Fatty Liver Index	267	4.44 (11.19)	6.13 (15.49)	5.74 (13.78)	4.58 (11.61)	3.88 (9.67)	0.125	0.131
**Inflammation**								
High-sensitivity CRP (mg/L)	259	1.55 (2.94)	1.36 (1.57)	1.86 (3.87)	1.60 (3.25)	1.44 (2.42)	0.275	0.876
High-sensitivity CRP (lab-specific)	263						**0.032***	
<1		1,227 (61%)	37 (64%)	151 (57%)	422 (59%)	617 (64%)		
1<3		556 (28%)	13 (22%)	72 (27%)	224 (31%)	247 (26%)		
≥3		218 (11%)	8 (14%)	40 (15%)	74 (10%)	96 (10%)		
**Hormonal markers**								

Serum total testosterone (nmol/L)	257	8.66 (9.44)	6.87 (8.02)	6.14 (8.04)	7.47 (8.85)	10.36 (9.98)	**<0.001*****	**<0.001*****
Serum SHBG (nmol/L)	256	49.63 (34.64)	48.75 (28.14)	48.61 (29.26)	50.67 (40.24)	49.19 (31.70)	0.807	0.907
Free testosterone (nmol/L)	258	1.67 (0.56)	1.65 (0.59)	1.66 (0.60)	1.66 (0.57)	1.69 (0.54)	0.727	0.385
Bioavailable testosterone (nmol/L)	258	4.20 (4.59)	3.52 (4.11)	3.14 (4.22)	3.64 (4.36)	4.96 (4.77)	**<0.001*****	**0.024***
Free androgen index	258	41.19 (14.61)	41.34 (15.60)	40.57 (15.83)	40.70 (14.77)	41.72 (14.07)	0.468	0.255
**Cardiometabolic risk indices**								
Framingham Risk Score	475	6.28 (4.69)	5.49 (3.31)	5.20 (3.73)	5.80 (4.28)	6.97 (5.17)	**<0.001*****	0.223
FINRISK Score	555	1.86 (1.46)	1.56 (0.92)	1.50 (1.11)	1.70 (1.26)	2.11 (1.66)	**<0.001*****	0.109
Metabolic syndrome (IDF criteria)	257	666 (33%)	20 (35%)	102 (39%)	243 (34%)	301 (31%)	0.126	0.888

Note. Values are presented as n (%) or mean (SD). *P*-values for differences across coffee consumption groups were derived using Welch's one-way ANOVA for continuous variables and Pearson's χ^2 test with Monte Carlo–simulated p-values (10,000 replicates) for categorical variables. Interaction *p*-values were calculated using linear models for continuous variables and logistic regression for binary categorical variables, including coffee group, sex, and their interaction term. Interaction analyses were not performed for multi-category categorical variables. *p*-values: *** <0.001; ** <0.01; * <0.05.

WHO, World Health Organization; BP, blood pressure; CRP, C-reactive protein; FAI, free androgen index; HOMA, homeostasis model assessment; IDF, International Diabetes Federation; OGTT, oral glucose tolerance test; SHBG, sex hormone–binding globulin.

Supplementary Table S2 Sensitivity analysis: anthropometrics and body composition by coffee consumption group after excluding extreme BMI values

Measure	N	Non-consumers	1–2 cups/d	3–4 cups/d	≥5 cups/d	*p*-value
BMI (kg/m^2)	2196	27.10 (4.63)	26.68 (4.47)	26.79 (4.30)	26.55 (3.99)	0.556
Waist circumference (cm)	1961	92.38 (14.30)	91.64 (13.27)	91.44 (12.60)	91.62 (12.34)	0.964
Hip circumference (cm)	1961	100.57 (9.98)	100.30 (8.89)	99.65 (8.82)	99.13 (7.95)	0.185
Waist–hip ratio	1961	0.92 (0.08)	0.91 (0.08)	0.92 (0.08)	0.92 (0.08)	0.206
Body fat percentage	1949	29.51 (9.38)	30.17 (8.86)	29.37 (9.04)	26.89 (8.56)	<0.001***
Body fat mass (kg)	1949	24.24 (11.45)	23.95 (9.87)	23.21 (9.71)	21.65 (8.91)	<0.001***
Skeletal muscle mass (kg)	1949	31.13 (7.25)	30.11 (6.82)	30.49 (6.81)	32.42 (7.01)	<0.001***
Visceral fat area (cm^2)	1949	108.20 (43.76)	106.65 (39.17)	105.59 (38.56)	100.96 (36.44)	0.034*

Note. Values are presented as mean (SD). This sensitivity analysis excluded participants with BMI <18.5 or >40 kg/m^2. P-values were calculated using Welch's one-way ANOVA across coffee consumption groups. Statistical significance is indicated as $p < 0.05$*, $p < 0.01$**, and $p < 0.001$***.

Supplementary Material

European Journal of Nutrition – Luca Verroest et al., Luca.Verroest@Oulu.fi

Supplementary Table S3 Sex-stratified Spearman correlations between coffee intake and circulating metabolites

	Men						Women				
	N	ρ	95% CI	*p*	q		N	ρ	95% CI	*p*	q
**Lipoprotein subclasses**											
Extremely large VLDL particles	943	−0.047	[-0.111, 0.017]	0.149	0.367		1048	−0.008	[-0.068, 0.053]	0.808	0.930
IDL particles	943	0.094	[0.031, 0.157]	0.004	0.025	*	1048	0.014	[-0.046, 0.075]	0.645	0.883
Large HDL particles	943	0.032	[-0.032, 0.095]	0.331	0.606		1048	−0.004	[-0.065, 0.056]	0.886	0.972
Large LDL particles	943	0.094	[0.031, 0.157]	0.004	0.025	*	1048	0.006	[-0.055, 0.066]	0.849	0.954
Large VLDL particles	943	−0.068	[-0.131, -0.004]	0.037	0.137		1048	−0.015	[-0.076, 0.046]	0.627	0.864
Medium HDL particles	943	0.010	[-0.054, 0.074]	0.758	0.915		1048	−0.022	[-0.082, 0.039]	0.486	0.735
Medium LDL particles	943	0.089	[0.026, 0.152]	0.006	0.035	*	1048	0.002	[-0.058, 0.063]	0.942	0.991
Medium VLDL particles	943	−0.060	[-0.124, 0.003]	0.063	0.185		1048	−0.020	[-0.080, 0.041]	0.526	0.776
Small HDL particles	943	0.008	[-0.056, 0.072]	0.811	0.930		1048	−0.028	[-0.089, 0.032]	0.362	0.621
Small LDL particles	943	0.086	[0.022, 0.149]	0.008	0.042	*	1048	0.001	[-0.060, 0.061]	0.987	0.996
Small VLDL particles	943	−0.041	[-0.104, 0.023]	0.214	0.483		1048	−0.025	[-0.086, 0.035]	0.411	0.673
Very large HDL particles	943	0.053	[-0.011, 0.116]	0.105	0.274		1048	0.001	[-0.059, 0.062]	0.971	0.991
Very large VLDL particles	943	−0.060	[-0.123, 0.004]	0.067	0.193		1048	−0.005	[-0.065, 0.056]	0.883	0.972
Very small VLDL particles	943	0.056	[-0.008, 0.120]	0.085	0.227		1048	0.002	[-0.059, 0.062]	0.953	0.991
**Total cholesterol in lipoproteins**											
Cholesterol in IDL	943	0.108	[0.044, 0.171]	<0.001	0.015	*	1048	0.022	[-0.039, 0.082]	0.487	0.735
Cholesterol in L HDL	943	0.029	[-0.035, 0.093]	0.371	0.631		1048	−0.004	[-0.064, 0.057]	0.900	0.982

	Men						Women				
	N	ρ	95% CI	*p*	q		N	ρ	95% CI	*p*	q
Cholesterol in L LDL	943	0.102	[0.038, 0.165]	0.002	0.019	*	1048	0.012	[-0.049, 0.072]	0.699	0.915
Cholesterol in L VLDL	943	−0.058	[-0.122, 0.006]	0.074	0.207		1048	−0.006	[-0.066, 0.055]	0.856	0.954
Cholesterol in M HDL	943	0.011	[-0.053, 0.075]	0.729	0.915		1048	−0.019	[-0.079, 0.042]	0.539	0.785
Cholesterol in M LDL	943	0.098	[0.035, 0.161]	0.002	0.020	*	1048	0.010	[-0.050, 0.071]	0.736	0.915
Cholesterol in M VLDL	943	−0.042	[-0.106, 0.022]	0.197	0.462		1048	−0.018	[-0.078, 0.043]	0.564	0.810
Cholesterol in S HDL	943	0.057	[-0.007, 0.120]	0.081	0.220		1048	−0.011	[-0.072, 0.050]	0.721	0.915
Cholesterol in S LDL	943	0.099	[0.036, 0.162]	0.002	0.019	*	1048	0.014	[-0.047, 0.074]	0.656	0.892
Cholesterol in S VLDL	943	0.019	[-0.045, 0.082]	0.568	0.810		1048	−0.009	[-0.070, 0.051]	0.760	0.915
Cholesterol in XL HDL	943	0.061	[-0.003, 0.125]	0.060	0.185		1048	0.011	[-0.049, 0.072]	0.717	0.915
Cholesterol in XL VLDL	943	−0.044	[-0.107, 0.020]	0.181	0.434		1048	0.008	[-0.053, 0.069]	0.796	0.930
Cholesterol in XS VLDL	943	0.098	[0.035, 0.161]	0.003	0.020	*	1048	0.030	[-0.031, 0.090]	0.339	0.606
Cholesterol in XXL VLDL	943	−0.038	[-0.102, 0.026]	0.242	0.530		1048	−0.001	[-0.062, 0.059]	0.964	0.991
**Triacylglycerol in lipoproteins**											
TG in IDL	943	0.011	[-0.052, 0.075]	0.727	0.915		1048	−0.029	[-0.089, 0.032]	0.355	0.621
TG in L HDL	943	0.037	[-0.027, 0.101]	0.254	0.532		1048	−0.015	[-0.076, 0.045]	0.619	0.858
TG in L LDL	943	0.041	[-0.023, 0.104]	0.210	0.483		1048	−0.020	[-0.080, 0.041]	0.518	0.771
TG in L VLDL	943	−0.071	[-0.134, - 0.007]	0.030	0.120		1048	−0.017	[-0.078, 0.043]	0.577	0.818
TG in M HDL	943	−0.049	[-0.112, 0.015]	0.135	0.341		1048	−0.024	[-0.085, 0.036]	0.429	0.687

Supplementary Material

European Journal of Nutrition – Luca Verroest et al., Luca.Verroest@Oulu.fi

	Men						Women				
	N	ρ	95% CI	*p*	q		N	ρ	95% CI	*p*	q
TG in M LDL	943	0.040	[-0.024, 0.104]	0.216	0.483		1048	−0.023	[-0.084, 0.038]	0.455	0.713
TG in M VLDL	943	−0.067	[-0.131, -0.004]	0.038	0.137		1048	−0.022	[-0.083, 0.038]	0.469	0.719
TG in S HDL	943	−0.038	[-0.101, 0.026]	0.246	0.532		1048	−0.032	[-0.092, 0.029]	0.308	0.604
TG in S LDL	943	−0.010	[-0.074, 0.053]	0.750	0.915		1048	−0.033	[-0.093, 0.028]	0.290	0.585
TG in S VLDL	943	−0.063	[-0.127, 0.000]	0.052	0.171		1048	−0.032	[-0.093, 0.028]	0.298	0.590
TG in XL HDL	943	0.043	[-0.021, 0.107]	0.187	0.444		1048	−0.030	[-0.090, 0.031]	0.336	0.606
TG in XL VLDL	943	−0.063	[-0.126, 0.001]	0.053	0.171		1048	−0.008	[-0.068, 0.053]	0.800	0.930
TG in XS VLDL	943	−0.028	[-0.092, 0.036]	0.390	0.651		1048	−0.033	[-0.093, 0.028]	0.293	0.586
TG in XXL VLDL	943	−0.048	[-0.111, 0.016]	0.143	0.356		1048	−0.009	[-0.069, 0.052]	0.772	0.921
**Lipoprotein particle size**											
HDL particle size	943	0.031	[-0.033, 0.095]	0.336	0.606		1048	0.000	[-0.060, 0.061]	0.994	0.996
LDL particle size	943	0.012	[-0.052, 0.075]	0.723	0.915		1048	0.013	[-0.048, 0.073]	0.681	0.915
VLDL particle size	943	−0.085	[-0.148, -0.021]	0.009	0.045	*	1048	−0.018	[-0.078, 0.043]	0.567	0.810
**Composite lipid measures**											
Esterified cholesterol	942	0.091	[0.027, 0.154]	0.005	0.032	*	1047	0.002	[-0.059, 0.062]	0.960	0.991
Free cholesterol	942	0.094	[0.030, 0.157]	0.004	0.026	*	1047	−0.001	[-0.061, 0.060]	0.986	0.996
HDL cholesterol	943	0.035	[-0.029, 0.098]	0.286	0.584		1048	−0.010	[-0.070, 0.051]	0.757	0.915
LDL cholesterol	943	0.100	[0.037, 0.163]	0.002	0.019	*	1048	0.012	[-0.049, 0.072]	0.704	0.915
Remnant cholesterol	943	0.041	[-0.023, 0.104]	0.212	0.483		1048	0.000	[-0.061, 0.060]	0.996	0.996

	Men						Women				
	N	ρ	95% CI	*p*	q		N	ρ	95% CI	*p*	q
Serum triglycerides	943	−0.058	[-0.122, 0.006]	0.074	0.207		1048	−0.028	[-0.089, 0.032]	0.360	0.621
Total cholesterol in HDL2	943	0.030	[-0.034, 0.094]	0.357	0.621		1048	−0.011	[-0.072, 0.050]	0.722	0.915
Total cholesterol in HDL3	943	0.075	[0.011, 0.138]	0.021	0.087		1048	0.001	[-0.059, 0.062]	0.971	0.991
Total cholesterol in VLDL	943	−0.008	[-0.072, 0.055]	0.796	0.930		1048	−0.012	[-0.072, 0.049]	0.710	0.915
Total serum cholesterol	943	0.090	[0.027, 0.153]	0.006	0.033	*	1048	0.001	[-0.059, 0.062]	0.967	0.991
Triglycerides in HDL	943	−0.037	[-0.101, 0.027]	0.256	0.532		1048	−0.044	[-0.104, 0.017]	0.156	0.378
Triglycerides in LDL	943	0.033	[-0.031, 0.096]	0.317	0.606		1048	−0.024	[-0.085, 0.036]	0.431	0.687
Triglycerides in VLDL	943	−0.066	[-0.129, -0.002]	0.043	0.152		1048	−0.023	[-0.083, 0.038]	0.467	0.719
**Phosphoglycerides & cholines**											
Phosphatidylcholine & cholines	942	0.063	[-0.001, 0.126]	0.053	0.171		1047	−0.012	[-0.072, 0.049]	0.699	0.915
Sphingomyelins	942	0.096	[0.032, 0.158]	0.003	0.024	*	1047	0.007	[-0.054, 0.067]	0.830	0.941
TG to phosphoglycerides ratio	942	−0.088	[-0.151, -0.024]	0.007	0.038	*	1047	−0.047	[-0.107, 0.014]	0.131	0.333
Total cholines	942	0.082	[0.019, 0.145]	0.011	0.053		1047	−0.002	[-0.063, 0.058]	0.945	0.991
Total phosphoglycerides	942	0.074	[0.010, 0.137]	0.023	0.094		1047	0.003	[-0.058, 0.064]	0.921	0.991
**Apolipoproteins**											
ApoB/ApoA1 ratio	943	0.006	[-0.058, 0.070]	0.853	0.954		1048	0.004	[-0.057, 0.064]	0.905	0.983
Apolipoprotein A1	943	0.032	[-0.031, 0.096]	0.319	0.606		1048	−0.030	[-0.090, 0.031]	0.331	0.606
Apolipoprotein B	943	0.027	[-0.037, 0.090]	0.411	0.673		1048	−0.009	[-0.069, 0.052]	0.778	0.923
**Fatty acids**											

	Men						Women					
	**N**	**ρ**	**95% CI**	***p***	**q**		**N**	**ρ**	**95% CI**	***p***	**q**	
Docosahexaenoic acid (DHA)	937	−0.093	[-0.156, -0.029]	0.004	0.027	*	1044	−0.098	[-0.157, -0.037]	0.002	0.019	*
Estimated degree of unsaturation	937	−0.054	[-0.117, 0.010]	0.101	0.268		1044	−0.091	[-0.151, -0.031]	0.003	0.024	*
Linoleic acid (LA)	937	0.068	[0.004, 0.131]	0.038	0.137		1044	−0.009	[-0.070, 0.051]	0.763	0.915	
Monounsaturated fatty acids	937	−0.033	[-0.097, 0.031]	0.313	0.606		1044	−0.023	[-0.084, 0.038]	0.458	0.713	
Omega-3 fatty acids	937	−0.112	[-0.174, -0.048]	<0.001	0.013	*	1044	−0.109	[-0.168, -0.049]	<0.001	0.012	*
Omega-6 fatty acids	937	0.057	[-0.007, 0.121]	0.080	0.220		1044	−0.016	[-0.077, 0.044]	0.598	0.841	
Polyunsaturated fatty acids	937	0.026	[-0.038, 0.090]	0.428	0.687		1044	−0.036	[-0.097, 0.024]	0.242	0.530	
Saturated fatty acids	937	0.027	[-0.037, 0.091]	0.412	0.673		1044	−0.011	[-0.071, 0.050]	0.734	0.915	
Total fatty acids	937	−0.001	[-0.065, 0.063]	0.971	0.991		1044	−0.027	[-0.088, 0.033]	0.377	0.635	
**Fatty acids (composition, %)**												
DHA / total FA (%)	937	−0.103	[-0.166, -0.039]	0.002	0.019	*	1044	−0.097	[-0.157, -0.037]	0.002	0.019	*
LA / total FA (%)	937	0.071	[0.007, 0.134]	0.031	0.120		1044	0.010	[-0.051, 0.071]	0.746	0.915	
MUFA / total FA (%)	937	−0.081	[-0.144, -0.017]	0.014	0.060		1044	−0.020	[-0.080, 0.041]	0.528	0.776	
Omega-3 / total FA (%)	937	−0.127	[-0.190, -0.064]	<0.001	0.006	**	1044	−0.113	[-0.173, -0.053]	<0.001	0.010	**
Omega-6 / total FA (%)	937	0.061	[-0.003, 0.124]	0.063	0.185		1044	0.001	[-0.059, 0.062]	0.967	0.991	
PUFA / total FA (%)	937	0.013	[-0.051, 0.077]	0.690	0.915		1044	−0.035	[-0.096, 0.025]	0.252	0.532	
SFA / total FA (%)	937	0.103	[0.039, 0.166]	0.002	0.019	*	1044	0.058	[-0.003, 0.118]	0.062	0.185	
**Amino acids**												
Alanine	942	−0.147	[-0.209, -0.084]	<0.001	0.001	**	1048	−0.081	[-0.141, -0.020]	0.009	0.044	*

	Men						Women					
	N	ρ	95% CI	*p*	q		N	ρ	95% CI	*p*	q	
Glutamine	939	0.035	[-0.029, 0.099]	0.286	0.584		1048	0.094	[0.034, 0.154]	0.002	0.019	*
Glycine	936	0.061	[-0.003, 0.125]	0.060	0.185		1044	0.106	[0.046, 0.166]	<0.001	0.013	*
Histidine	942	0.032	[-0.032, 0.096]	0.322	0.606		1046	−0.016	[-0.076, 0.045]	0.608	0.849	
Isoleucine	939	−0.141	[-0.203, - 0.078]	<0.001	0.001	**	1048	−0.095	[-0.154, - 0.034]	0.002	0.019	*
Leucine	941	−0.118	[-0.181, - 0.054]	<0.001	0.010	**	1048	−0.107	[-0.167, - 0.047]	<0.001	0.013	*
Phenylalanine	942	−0.064	[-0.127, 0.000]	0.049	0.170		1048	−0.047	[-0.107, 0.014]	0.128	0.330	
Tyrosine	942	−0.101	[-0.164, - 0.038]	0.002	0.019	*	1047	−0.084	[-0.144, - 0.023]	0.007	0.036	*
Valine	938	−0.106	[-0.169, - 0.042]	0.001	0.018	*	1048	−0.083	[-0.143, - 0.022]	0.007	0.038	*
**Glycolysis-related**												
Glucose	942	−0.037	[-0.101, 0.027]	0.255	0.532		1047	−0.060	[-0.120, 0.001]	0.053	0.171	
Glycerol	934	−0.102	[-0.165, - 0.038]	0.002	0.019	*	1036	−0.116	[-0.175, - 0.055]	<0.001	0.010	**
Lactate	942	−0.061	[-0.124, 0.003]	0.063	0.185		1048	−0.060	[-0.120, 0.001]	0.054	0.171	
Pyruvate	941	−0.082	[-0.146, - 0.019]	0.011	0.053		1048	−0.077	[-0.137, - 0.016]	0.013	0.059	
**Ketone bodies**												
3-hydroxybutyrate	938	−0.022	[-0.086, 0.042]	0.496	0.744		1047	−0.085	[-0.145, - 0.024]	0.006	0.035	*
Acetoacetate	940	−0.005	[-0.069, 0.059]	0.872	0.967		1048	−0.067	[-0.127, - 0.006]	0.031	0.120	
**Inflammation (glycoproteins)**												
Glycoprotein acetyls	941	−0.031	[-0.095, 0.033]	0.337	0.606		1048	−0.029	[-0.089, 0.032]	0.353	0.621	
**Miscellaneous**												

	Men					Women					
	N	ρ	95% CI	*p*	q	N	ρ	95% CI	*p*	q	
Acetate	942	−0.008	[-0.072, 0.056]	0.811	0.930	1048	−0.007	[-0.067, 0.054]	0.825	0.940	
Albumin	943	0.002	[-0.061, 0.066]	0.939	0.991	1048	−0.023	[-0.084, 0.037]	0.451	0.713	
Citrate	941	−0.078	[-0.141, -0.014]	0.016	0.070	1048	−0.103	[-0.162, -0.042]	<0.001	0.015	*
Creatinine	941	−0.079	[-0.142, -0.015]	0.016	0.069	1047	−0.065	[-0.126, -0.005]	0.034	0.130	

Note. Spearman rank correlations were calculated separately in men and women between habitual coffee intake (cups/day) and NMR-quantified circulating metabolites. Results are presented as available N, correlation coefficient (ρ), 95% confidence interval, raw *p*-value, and FDR-adjusted q-value. Sample sizes vary across metabolites due to variable-specific missing data. Asterisks indicate FDR-adjusted significance: q < 0.05*, q < 0.01**, and q < 0.001***.

Supplementary Material

European Journal of Nutrition – Luca Verroest et al., Luca.Verroest@Oulu.fi

Supplementary Table S4 Sex-stratified Spearman correlations between coffee intake and cardiometabolic risk factors

	Men					Women				
	**N**	**ρ**	**95% CI**	***p***		**N**	**ρ**	**95% CI**	***p***	
**Sociodemographics & Lifestyle**										
Alcohol consumption (g/day)	1025	−0.033	[-0.094, 0.028]	0.292		1143	−0.048	[-0.105, 0.010]	0.107	
**Anthropometrics**										
Body mass index (BMI)	1069	−0.046	[-0.106, 0.014]	0.132		1177	−0.008	[-0.066, 0.049]	0.773	
Hip circumference (cm)	945	−0.049	[-0.112, 0.015]	0.133		1057	−0.019	[-0.079, 0.042]	0.542	
Waist–hip ratio	945	−0.058	[-0.121, 0.006]	0.076		1057	−0.095	[-0.154, 0.035]	- 0.002	**
Waist circumference (cm)	946	−0.055	[-0.119, 0.008]	0.089		1057	−0.060	[-0.119, 0.001]	0.053	
**Body composition**										
Body fat mass (kg)	936	−0.070	[-0.133, 0.006]	- 0.033	*	1055	−0.046	[-0.106, 0.015]	0.139	
Body fat percentage	936	−0.083	[-0.147, 0.019]	- 0.011	*	1055	−0.062	[-0.122, 0.002]	- 0.044	*
Skeletal muscle mass (kg)	936	0.006	[-0.058, 0.070]	0.856		1055	0.025	[-0.035, 0.085]	0.415	
Visceral fat area (cm²)	936	−0.048	[-0.112, 0.016]	0.139		1055	−0.027	[-0.087, 0.033]	0.377	
**Blood pressure (mmHg)**										
Mean diastolic BP	950	0.021	[-0.042, 0.085]	0.514		1059	−0.014	[-0.075, 0.046]	0.641	
Mean systolic BP	950	0.058	[-0.005, 0.122]	0.072		1059	0.002	[-0.058, 0.062]	0.952	
**Fasting serum lipids (mmol/L)**										
HDL cholesterol	954	0.050	[-0.013, 0.113]	0.122		1061	0.005	[-0.055, 0.065]	0.870	
LDL cholesterol	954	0.072	[0.008, 0.135]	0.026	*	1061	−0.006	[-0.066, 0.054]	0.844	

	Men						Women					
	N	ρ	95% CI		*p*		N	ρ	95% CI		*p*	
Total cholesterol	952	0.052	[-0.011, 0.116]		0.106		1061	−0.013	[-0.073, 0.047]		0.677	
Triglycerides	954	−0.075	[-0.138, 0.012]	-	0.020	*	1061	−0.044	[-0.104, 0.016]		0.150	
**Fasting values**												
Fasting plasma glucose	929	−0.034	[-0.098, 0.030]		0.297		1049	−0.049	[-0.110, 0.011]		0.109	
Fasting serum insulin	938	−0.095	[-0.158, 0.031]	-	0.004	**	1051	−0.042	[-0.102, 0.019]		0.174	
**OGTT (mmol/L)**												
Plasma glucose at 0 min	847	0.009	[-0.058, 0.077]		0.788		939	−0.060	[-0.124, 0.004]		0.066	
Plasma glucose at 120 min	832	−0.060	[-0.128, 0.008]		0.083		927	−0.048	[-0.112, 0.017]		0.146	
Plasma glucose at 30 min	828	−0.062	[-0.129, 0.006]		0.075		926	−0.023	[-0.087, 0.042]		0.492	
Plasma glucose at 60 min	828	−0.067	[-0.135, 0.001]		0.053		920	−0.026	[-0.090, 0.039]		0.435	
Serum insulin at 0 min	847	−0.055	[-0.122, 0.012]		0.109		941	−0.083	[-0.146, 0.020]	-	0.011	*
Serum insulin at 120 min	833	−0.153	[-0.219, 0.086]	-	<0.001	***	930	−0.034	[-0.098, 0.031]		0.306	
Serum insulin at 30 min	828	−0.075	[-0.142, 0.007]	-	0.031	*	926	−0.007	[-0.072, 0.057]		0.827	
Serum insulin at 60 min	831	−0.127	[-0.194, 0.060]	-	<0.001	***	924	−0.011	[-0.075, 0.054]		0.742	
**OGTT derived indices**												
Glucose AUC (0–180 min)	813	−0.071	[-0.139, 0.002]	-	0.043	*	895	−0.038	[-0.103, 0.027]		0.254	
QUICKI (OGTT-derived insulin sensitivity)	839	0.052	[-0.016, 0.119]		0.132		925	0.088	[0.024, 0.152]		0.007	**
**Insulin sensitivity/resistance**												
HOMA2 insulin resistance	898	−0.106	[-0.171, 0.041]	-	0.001	**	1004	−0.048	[-0.110, 0.014]		0.127	

Supplementary Material

European Journal of Nutrition – Luca Verroest et al., Luca.Verroest@Oulu.fi

	Men					Women				
	N	ρ	95% CI	*p*		N	ρ	95% CI	*p*	
HOMA2 insulin sensitivity	898	0.106	[0.041, 0.171]	0.001	**	1004	0.048	[-0.014, 0.110]	0.127	
HOMA2 β-cell function	898	−0.090	[-0.155, 0.025] -	0.007	**	1004	−0.024	[-0.086, 0.038]	0.441	
**Liver & kidney markers**										
Alanine aminotransferase (U/L)	954	−0.082	[-0.145, 0.019] -	0.011	*	1061	−0.080	[-0.139, 0.020] -	0.009	**
Aspartate aminotransferase (U/L)	951	−0.047	[-0.110, 0.017]	0.147		1061	−0.050	[-0.110, 0.010]	0.104	
Fatty Liver Index	943	−0.082	[-0.145, 0.018] -	0.012	*	1054	−0.047	[-0.107, 0.013]	0.127	
Gamma-glutamyl transferase (U/L)	952	−0.087	[-0.149, 0.023] -	0.007	**	1061	−0.050	[-0.110, 0.010]	0.103	
Serum albumin (g/L)	954	0.009	[-0.055, 0.072]	0.782		1061	0.021	[-0.040, 0.081]	0.504	
**Inflammation**										
High-sensitivity CRP (mg/L)	950	0.019	[-0.044, 0.083]	0.551		1055	−0.087	[-0.146, 0.026] -	0.005	**
**Hormonal markers**										
Bioavailable testosterone (nmol/L)	949	0.121	[0.058, 0.184]	<0.001	***	1057	−0.019	[-0.080, 0.041]	0.528	
Free androgen index	949	−0.143	[-0.205, 0.080] -	<0.001	***	1057	−0.052	[-0.112, 0.008]	0.089	
Free testosterone (nmol/L)	949	−0.151	[-0.213, 0.088] -	<0.001	***	1057	−0.055	[-0.115, 0.005]	0.073	
Serum SHBG (nmol/L)	950	0.169	[0.106, 0.230]	<0.001	***	1058	0.056	[-0.004, 0.116]	0.069	
Serum total testosterone (nmol/L)	949	0.179	[0.116, 0.240]	<0.001	***	1058	0.050	[-0.010, 0.110]	0.102	
**CVD risk scores**										
FINRISK Score	798	0.106	[0.037, 0.175]	0.003	**	911	0.100	[0.035, 0.164]	0.003	**
Framingham Risk Score	845	0.080	[0.013, 0.147]	0.020	*	944	0.064	[0.000, 0.127]	0.051	

	Men				Women			
	**N**	**ρ**	**95% CI**	***p***	**N**	**ρ**	**95% CI**	***p***

Note. Spearman rank correlations were calculated separately in men and women between habitual coffee intake (cups/day) and cardiometabolic and hormonal markers. Results are presented as available N, correlation coefficient (ρ), 95% confidence interval, and *p*-value. Sample sizes vary across variables due to variable-specific missing data. Asterisks indicate nominal statistical significance based on raw *p*-values: * < 0.05, ** < 0.01, and *** < 0.001.

European Journal of Nutrition – Luca Verroest et al., Luca.Verroest@Oulu.fi

Supplementary Table S5. Exploratory GAM assessment of non-linearity in coffee–hormone associations

Hormonal marker	N	edf	*p* for smooth term	AIC, linear model	AIC, GAM	ΔAIC	Interpretation
**Men**							
Serum total testosterone (nmol/L)	896	2.25	<0.001	5758.9	5755.5	-3.3	Modest non-linearity
Serum SHBG (nmol/L)	897	1.11	<0.001	7209.1	7209.3	0.2	Approximately linear
Free testosterone (nmol/L)	896	1.00	<0.001	744.3	744.3	0.0	Approximately linear
Bioavailable testosterone (nmol/L)	896	2.14	0.004	4292.4	4290.0	-2.4	Modest non-linearity
Free androgen index	896	1.00	<0.001	6562.8	6562.8	0.0	Approximately linear
**Women**							
Serum total testosterone (nmol/L)	938	1.00	0.845	1394.2	1394.2	0.0	Approximately linear
Serum SHBG (nmol/L)	938	1.00	0.016	9587.9	9587.9	0.0	Approximately linear
Free testosterone (nmol/L)	937	1.00	0.015	1067.5	1067.5	0.0	Approximately linear
Bioavailable testosterone (nmol/L)	937	1.00	0.097	212.0	212.0	0.0	Approximately linear
Free androgen index	937	1.00	0.026	7163.5	7163.5	0.0	Approximately linear

Note**.** Generalized additive models (GAMs) included a smooth term for coffee intake and were adjusted for body mass index (BMI), education, smoking status, physical activity, and alcohol intake. Female analyses excluded women with reported polycystic ovary syndrome (PCOS) or missing PCOS status. edf, estimated degrees of freedom; values close to 1 indicate an approximately linear association, whereas values >1 suggest curvature. AIC, Akaike information criterion; ΔAIC, difference in AIC, calculated as AIC(GAM) − AIC(linear model). Negative ΔAIC values indicate better fit of the GAM.

European Journal of Nutrition – Luca Verroest et al., Luca.Verroest@Oulu.fi

Supplementary Table S6 Sex-stratified linear regression associations between coffee intake and hormonal markers

Hormonal marker	Unadjusted			BMI-adjusted			Fully adjusted		
	N	β (95% CI)	*p*	N	β (95% CI)	*p*	N	β (95% CI)	*p*
**Men**									
Serum total testosterone (nmol/L)	949	0.348 (0.222, 0.474)	<0.001	948	0.302 (0.187, 0.418)	<0.001	896	0.288 (0.170, 0.407)	<0.001
Serum SHBG (nmol/L)	950	0.697 (0.430, 0.964)	<0.001	949	0.624 (0.370, 0.878)	<0.001	897	0.570 (0.306, 0.834)	<0.001
Free testosterone (nmol/L)	949	-0.017 (-0.024, -0.010)	<0.001	948	-0.016 (-0.023, -0.009)	<0.001	896	-0.014 (-0.021, -0.007)	<0.001
Bioavailable testosterone (nmol/L)	949	0.098 (0.044, 0.152)	<0.001	948	0.081 (0.030, 0.132)	0.002	896	0.079 (0.027, 0.131)	0.003
Free androgen index	949	-0.434 (-0.620, -0.249)	<0.001	948	-0.392 (-0.570, -0.213)	<0.001	896	-0.357 (-0.542, -0.172)	<0.001
**Women**									
Serum total testosterone (nmol/L)	949	0.000 (-0.013, 0.012)	0.983	949	0.000 (-0.013, 0.012)	0.988	938	-0.001 (-0.014, 0.012)	0.843
Serum SHBG (nmol/L)	949	1.038 (0.006, 2.071)	0.049	949	1.151 (0.153, 2.149)	0.024	938	1.272 (0.244, 2.301)	0.015
Free testosterone (nmol/L)	948	-0.010 (-0.021, 0.001)	0.083	948	-0.012 (-0.022, -0.001)	0.027	937	-0.014 (-0.025, -0.003)	0.015
Bioavailable testosterone (nmol/L)	948	-0.005 (-0.011, 0.002)	0.165	948	-0.005 (-0.012, 0.002)	0.134	937	-0.006 (-0.013, 0.001)	0.097
Free androgen index	948	-0.222 (-0.515, 0.071)	0.137	948	-0.268 (-0.541, 0.004)	0.053	937	-0.323 (-0.607, -0.040)	0.026

Note. Values are β-estimates with 95% confidence intervals from sex-stratified linear regression models. Estimates represent the change in each hormonal marker per additional cup of coffee per day. The unadjusted model included coffee intake only; the BMI-adjusted model included coffee intake and BMI; the fully adjusted model additionally included education, smoking status, physical activity, and alcohol intake. Female analyses excluded women with PCOS or missing PCOS status.
